# Supplementary material for: Sub-angstrom strain in high-entropy intermetallic boosts the oxygen reduction reaction in fuel cell cathodes
Source: Nat Commun. 2025 Aug 14;16:7547. doi: 10.1038/s41467-025-62725-7 (PMC12354711; doi:10.1038/s41467-025-62725-7)
Supplement: Supplementary file 1 — Supplementary Information [file 41467_2025_62725_MOESM1_ESM.pdf]

Supplementary Information

Sub-angstrom Strain in High-entropy Intermetallic Boosts the Oxygen Reduction Reaction in Fuel Cell Cathodes

Xueru Zhao[1]†, Hao Cheng[2]†, Lijun Wu[3], Qi Zhang[4], Xiaobo Chen[5], Nebojsa Marinkovic[6], Chenzhao Li[4], Sha Tan[1], Enyuan Hu[1], Lu Ma[7], Yimei Zhu[3], Jian Xie[4], and Kotaro Sasaki [1]*

†These authors contributed equally to this work.

*Corresponding Author. Email: ksasaki@bnl.gov

This file includes:

Supplementary Figs. 1 to 25
Supplementary Tables 1 to 12
Supplementary References

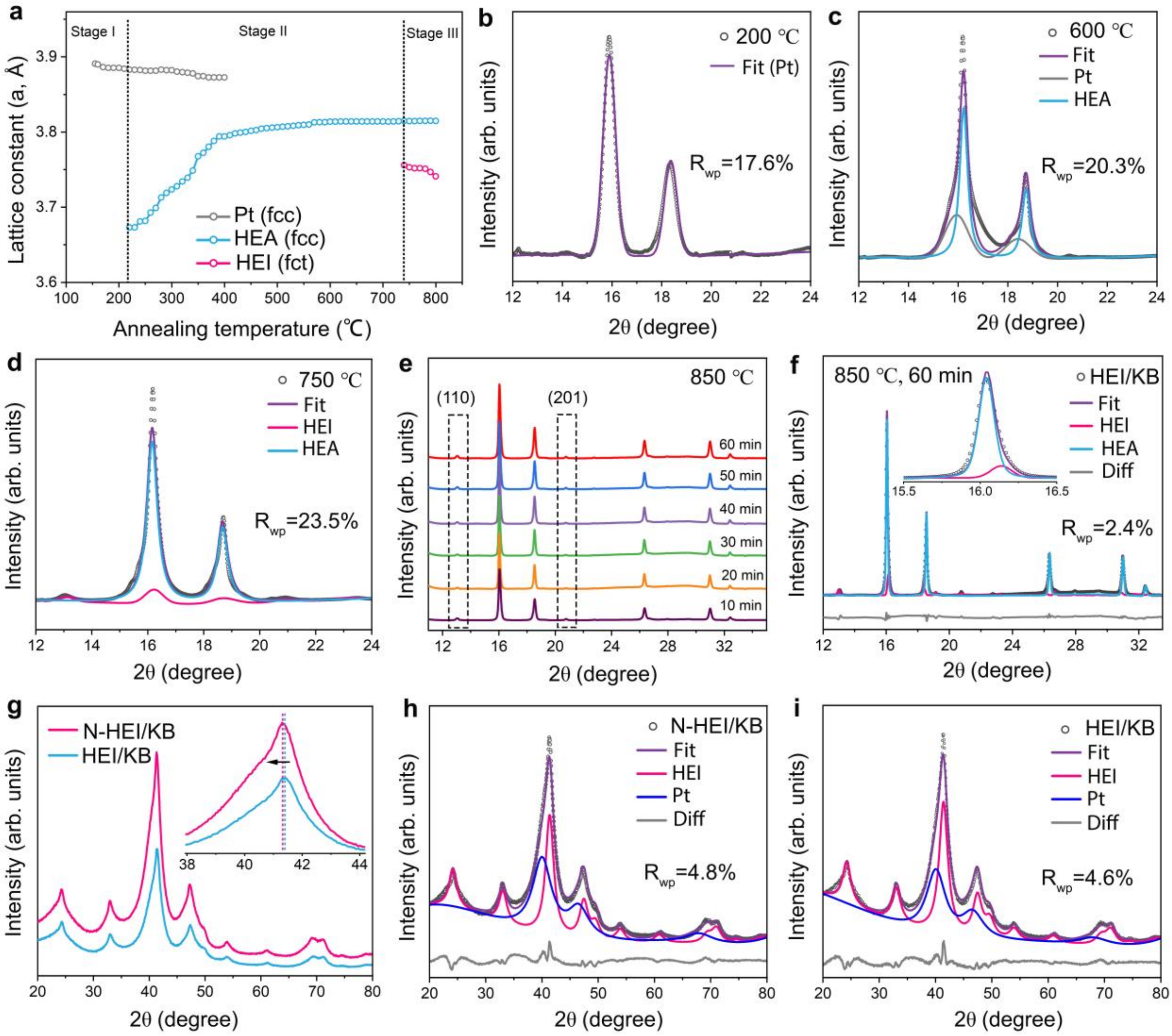

Supplementary Fig. 1 XRD results and Rietvled refinement analysis. a. Rietveld refinement analysis showing changes in lattice constant of the HEI nanoparticles as a function of annealing temperatures. b-d. Rietveld refinement of XRD patterns of HEI/KB at different annealing temperature: b. 200 °C, c. 600 °C, and d. 750 °C. e. In situ synchrotron XRD patterns of HEI/KB holding at 850 °C in 10% H_2/He gas stream (wavelength 0.6199 Å). f. Rietveld refinement of XRD patterns of HEI/KB holding at 850 °C for 60 min. g. XRD patterns of N-HEI/KB and HEI/KB catalysts (Cu Kα, wavelength 1.5406 Å). h-i. Rietveld refinement of XRD patterns of N-HEI/KB and HEI/KB in (g). The "Diff" curve represents the difference between the experimentally measured and the calculated XRD patterns, highlighting the quality of the Rietveld refinement fit.

Rietveld refinement was performed on the in situ XRD patterns collected at different thermal treatment stages to analyze the phase evolution using TOPAS software.[1] For stage I (<220 °C), the XRD patterns were refined using a cubic Pt phase with the Fm-3m space group. In stage II (220–740 °C), the patterns were fitted with a combination of cubic Pt (Fm-3m) and HEA (Fm-3m) phases. At stage III (>740 °C), the refinement was conducted using both the cubic HEA

phase (Fm-3m) and a tetragonal HEI phase with the P4/mmm space group, indicative of $L1_0$-type ordering. During high-temperature holding period (850 °C), the refinement was conducted using a two-phase model comprising the cubic HEA and the tetragonal HEI phase. Rietveld refinement of N-HEI/KB and HEI/KB catalysts (Cu Kα, wavelength 1.5406 Å) was also performed using a two-phase model comprising the cubic Pt phase and the tetragonal HEI phase. To simplify the refinement, Co, Ni, Fe, and Cu were grouped as a single transition metal component, and PtNi was used to represent the HEA/HEI structures for Rietveld refinements in TOPAS. Representative Rietveld refinement results from each stage were selected for illustration, as shown in Supplementary Fig. 1b-d.

The weighted profile R-factor (R_{wp}) values for the in situ Rietveld refinements ranged from 17% to 25%, indicating an acceptable level of agreement between the observed and calculated XRD profiles. It is worth noting that the diffraction peaks corresponding to the Pt phase became significantly weaker above 400 °C, resulting in increased uncertainty in the refined lattice parameters. Therefore, only the lattice constants of Pt obtained below 400 °C are presented in Supplementary Fig. 1a.

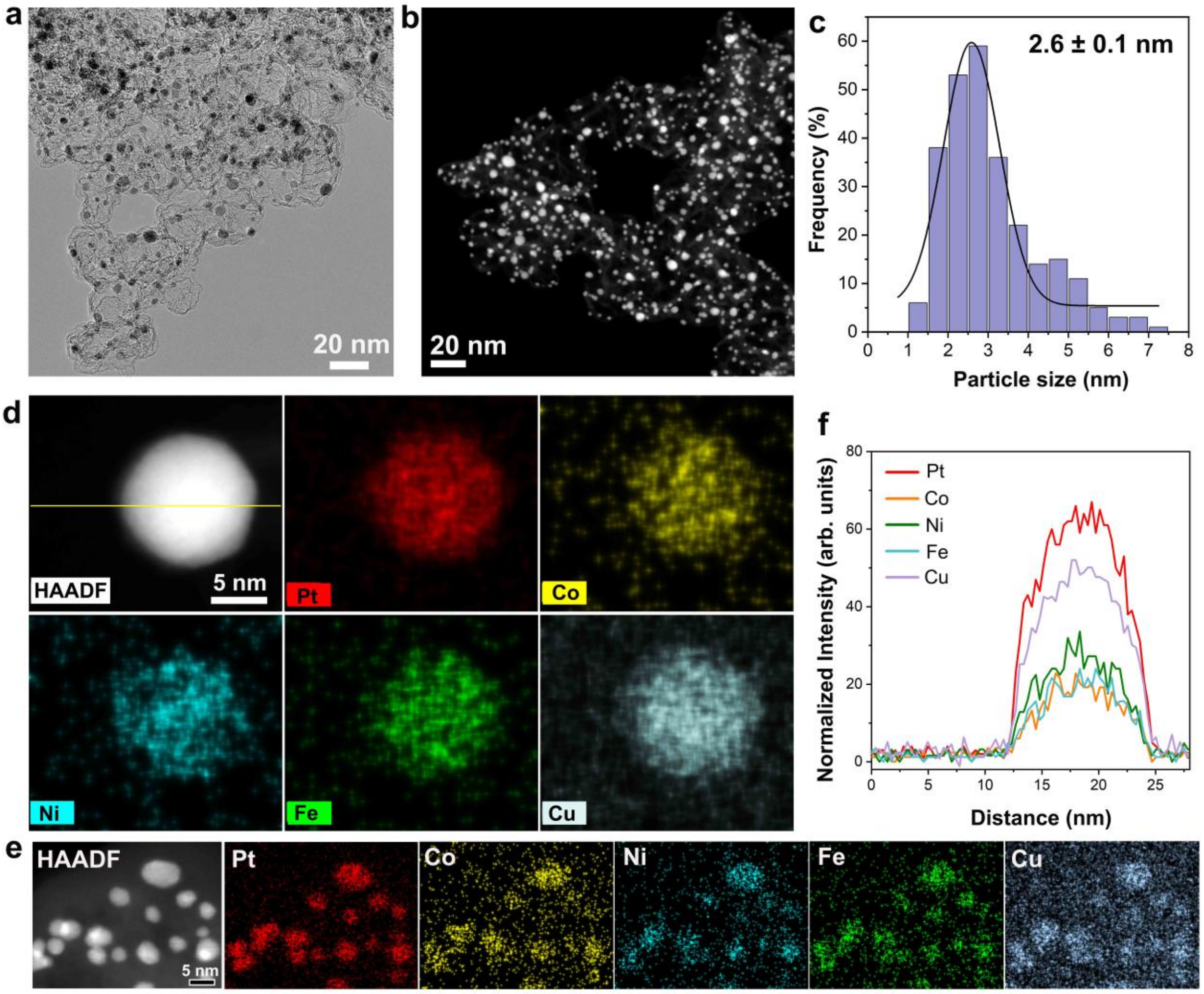

Supplementary Fig. 2 Morphology of HEI/KB catalyst. a. TEM image of HEI/KB catalyst. b. STEM-HAADF image of HEI/KB catalyst. c. The particle size distribution of the HEI/KB catalyst. d-e. STEM-HAADF image and the corresponding EDS elemental mappings of single and multiple HEI nanoparticles. f. STEM-EDS line scan profiles of HEI/KB along the yellow single line in d.

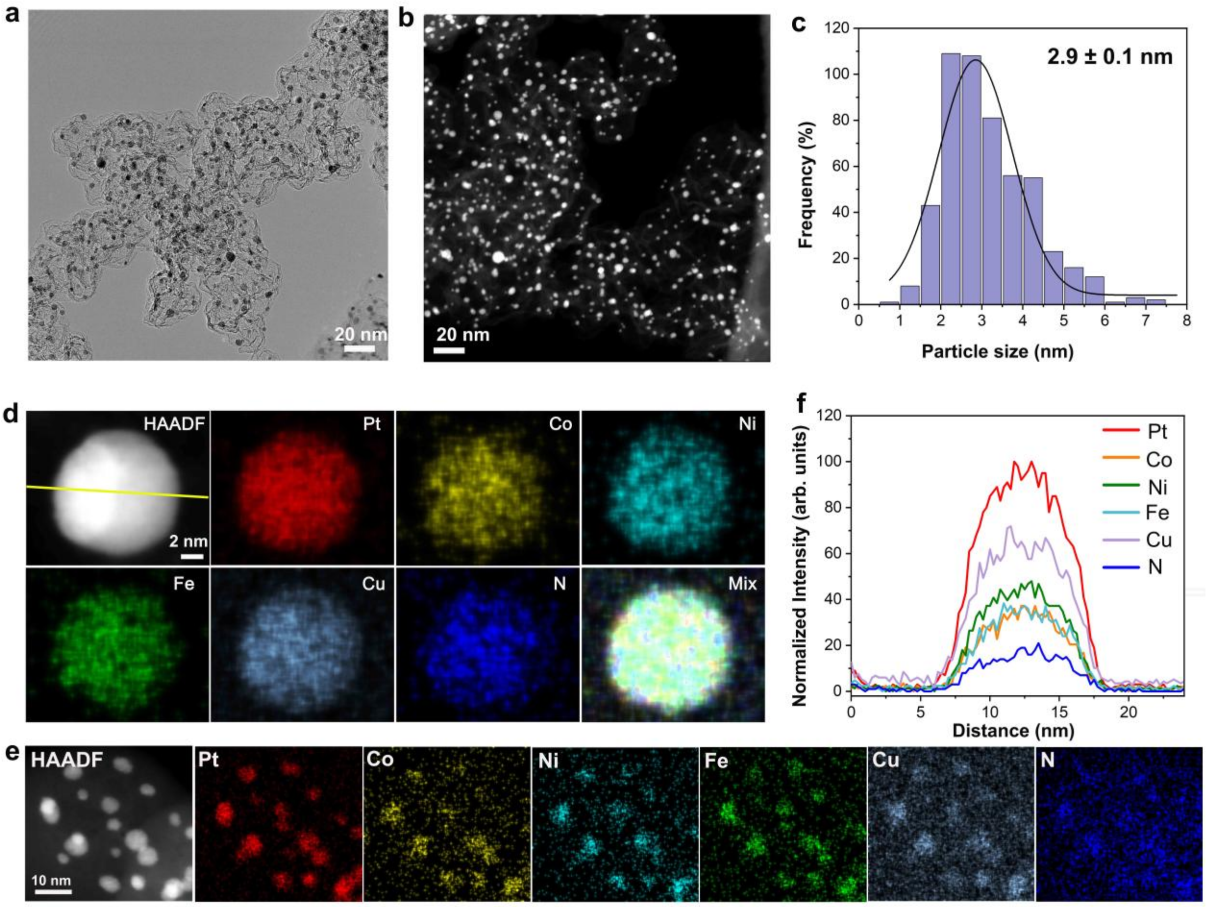

Supplementary Fig. 3 Morphology of N-HEI/KB catalyst. a. TEM image of the HEI/KB catalyst. b. STEM-HAADF image of N-HEI/KB catalyst. c. The particle size distribution of the N-HEI/KB catalyst. d-e. STEM-HAADF image and the corresponding EDS elemental mappings of single and multiple N-HEI nanoparticles. f. STEM-EDS line scan profiles of N-HEI/KB along the yellow single line in d.

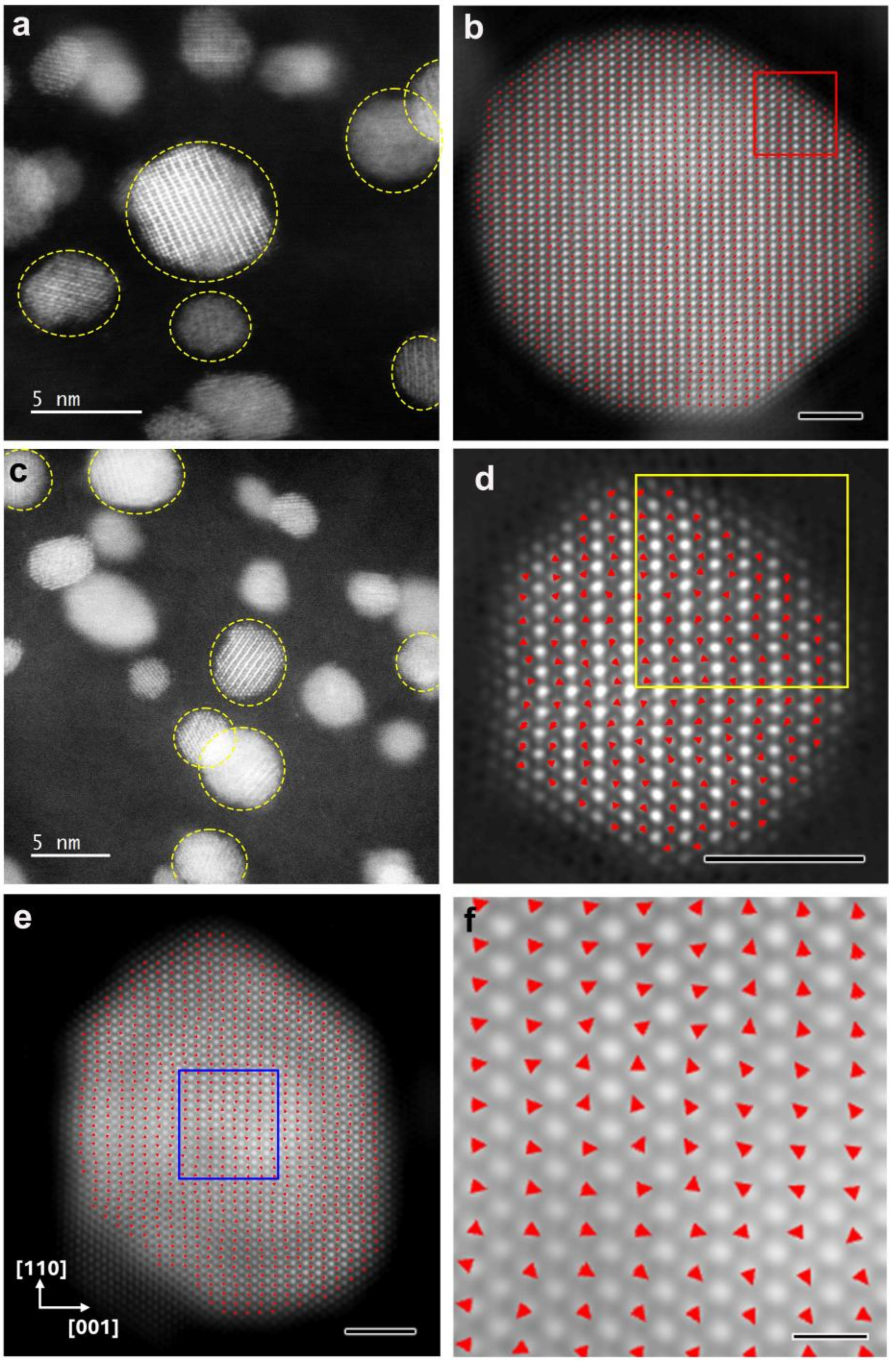

Supplementary Fig. 4 Atomic resolution STEM-HAADF images of N-HEI/KB and HEI/KB. a-b. Atomic resolution STEM-HAADF images of N-HEI/KB. c-d. Atomic resolution STEM-HAADF images of HEI/KB. e. Representative atomic-resolution STEM-HAADF image of PtNiN/KB particle viewed along [-110] direction. f. Enlarged image from the area marked by blue rectangle in e. The image is lightly filtered in frequency space by applying periodic mask to remove noise. The ordered phase in a and c are indicated by yellow dotted circles. The rectangles mark the areas in b and d shown in Fig. 1e-f. The arrows show the displacement of TM atoms from the center of four Pt atoms. The positions of Pt and TM are refined based on the second polynomial function. Scale bars are 2 nm for (b, d, e) and 0.5 nm for f.

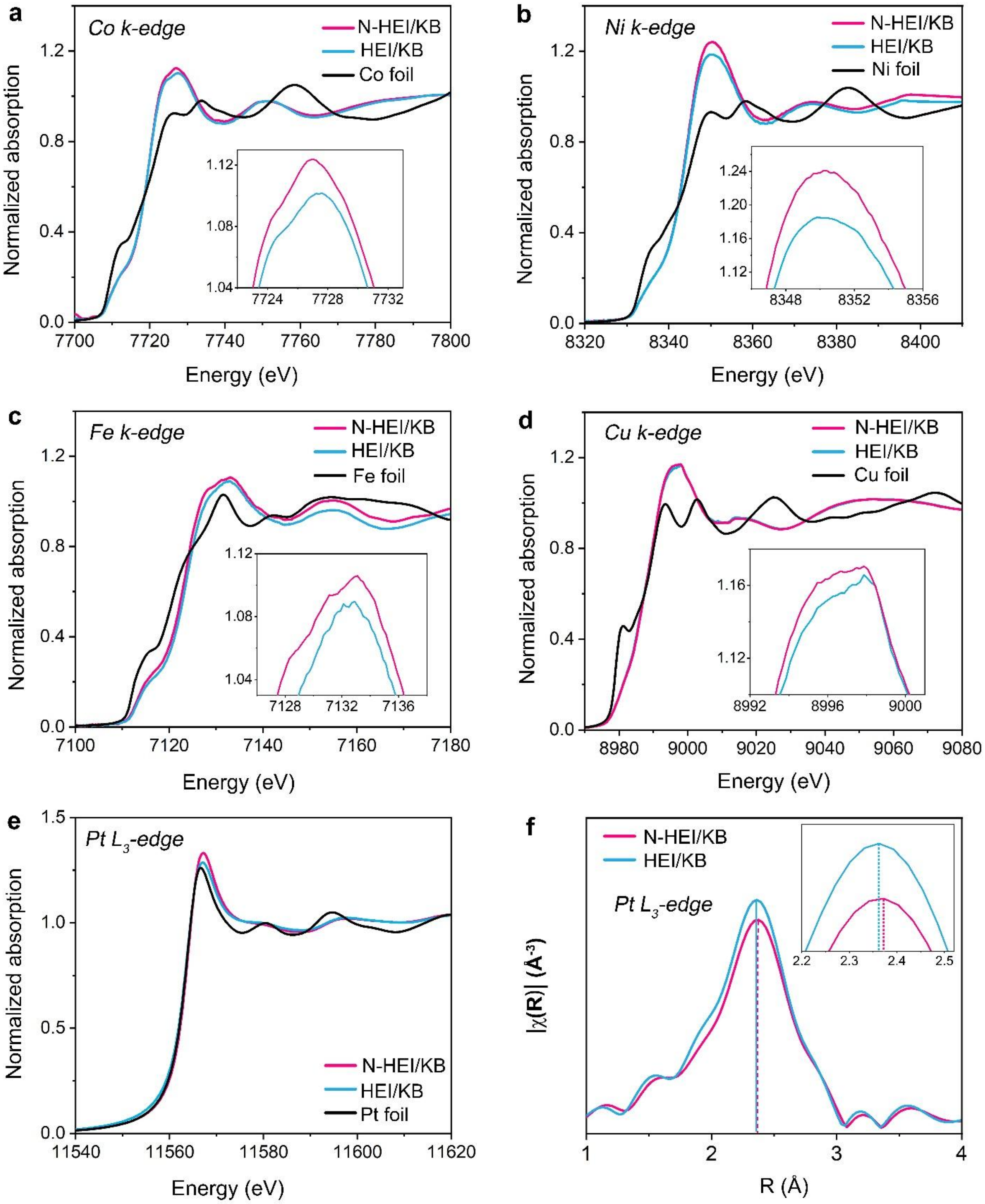

Supplementary Fig. 5 Characterization of electronic structure of N-HEI/KB. a. XANES spectra for the Co K-edge. b. XANES spectra for the Ni K-edge. c. XANES spectra for the Fe K-edge. d. XANES spectra for the Cu *k*-edge. e. XANES spectra for the Pt L_3-edge. f. Pt L_3-edge FT-EXAFS spectra of N-HEI/KB and HEI/KB catalysts.

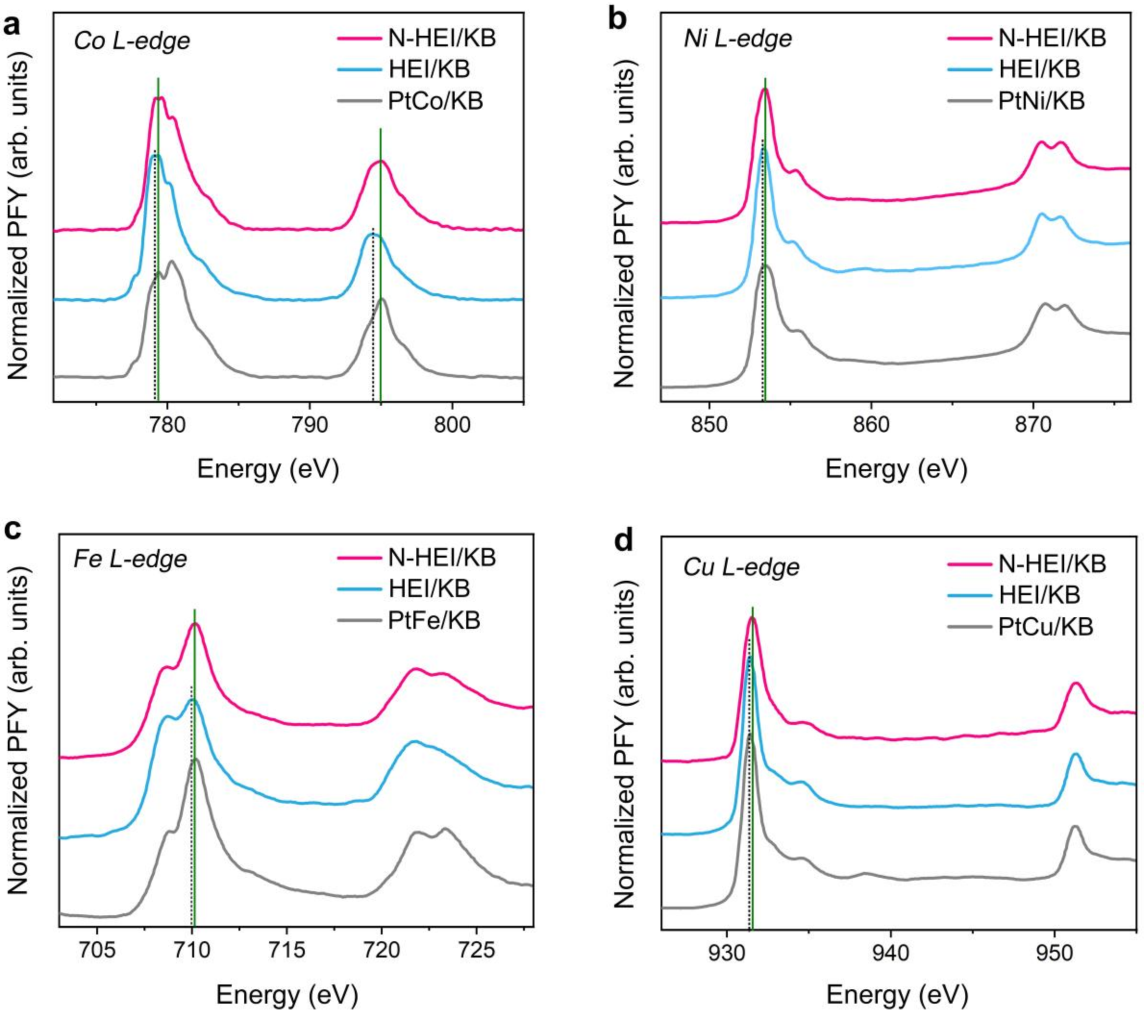

Supplementary Fig. 6 Soft XAS results of different samples. Soft XAS spectra of N-HEI/KB, HEI/KB, and corresponding binary Pt-based alloys: a. Co L-edges, b. Ni L-edges, c. Fe L-edges and d. Cu L-edges.

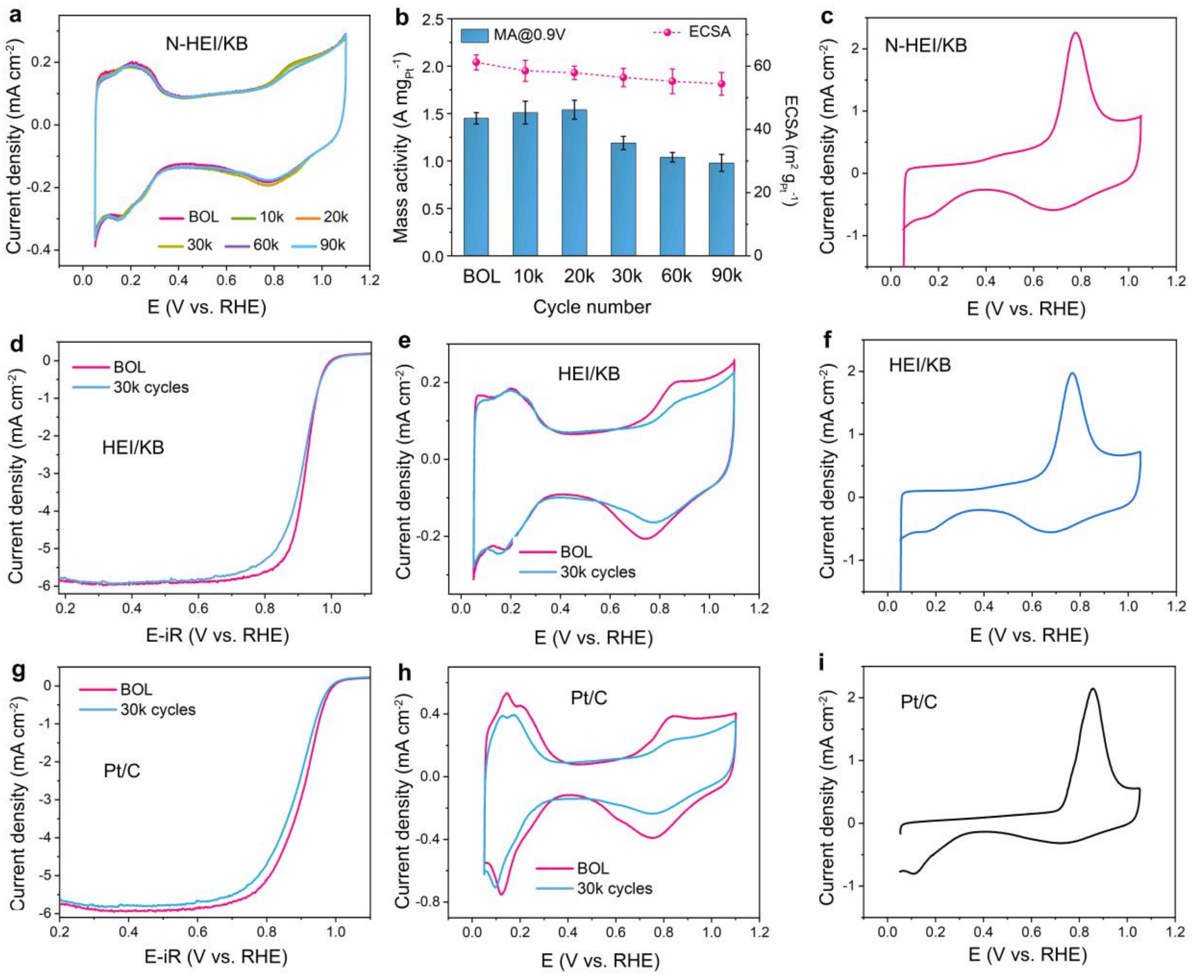

Supplementary Fig. 7 ORR performance of N-HEI/KB, HEI/KB, and commercial Pt/C in RDE testing. a. CV curves of N-HEA/C at beginning-of-life (BOL) and different ADT cycles. b. MA and ECSA of N-HEI/KB catalyst at BOL and different potential cycles. The error bars represent the standard deviation derived from three independent experimental measurements. c. CO stripping curve of N-HEI/KB. d-e. ORR polarization curves and CV curves of HEI/KB at BOL and ADT 30,000 cycles. f. CO stripping curve of HEI/KB. g-h. ORR polarization curves and CV curves of commercial Pt/C at BOL and ADT 30,000 cycles. i. CO stripping curve of commercial Pt/C. Notes: The ORR polarization curves were collected by using the LSV method in O_2-saturated 0.1 mol L^{-1} $HClO_4$ (pH = 1.0 $\pm$ 0.1) with a scanning rate of 10 mV s^{-1}. The CV curves were collected in Ar-saturated 0.1 M $HClO_4$ with a scanning rate of 20 mV s^{-1}. The CO stripping curves were collected in Ar-saturated 0.1 M $HClO_4$ with a scanning rate of 50 mV s^{-1}. The durability tests of all catalysts were conducted by cycling the potential between 0.6 and 0.95 V at a scanning rate of 100 mV s^{-1} in aerated 0.1 M $HClO_4$.

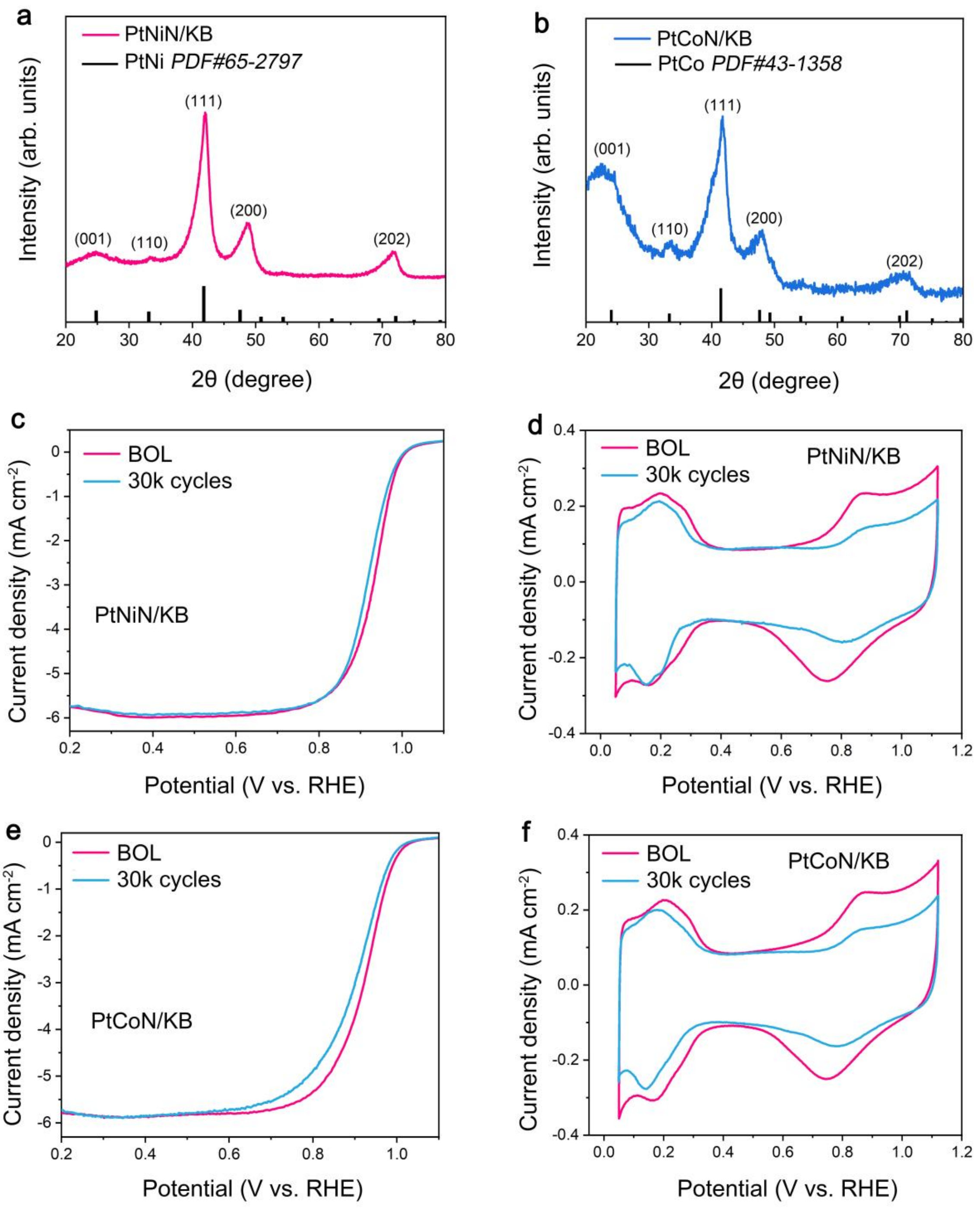

Supplementary Fig. 8 XRD and ORR performance of PtNiN/KB and PtCoN/KB in RDE testing. a. XRD patterns of PtNiN/KB. b. XRD patterns of PtCoN/KB (Cu Kα, wavelength 1.5406 Å). c-d. ORR polarization curves and CV curves of PtNiN/KB at BOL and ADT 30,000 cycles. e-f. ORR polarization curves and CV curves of PtCoN/KB at BOL and ADT 30,000 cycles. Notes: The ORR polarization curves were collected by using the LSV method in O_2-saturated 0.1 mol L^{-1} $HClO_4$ (pH = 1.0 ± 0.1) with a scanning rate of 10 mV s^{-1}. The CV curves were collected in Ar-saturated 0.1 M $HClO_4$ with a scanning rate of 20 mV s^{-1}.

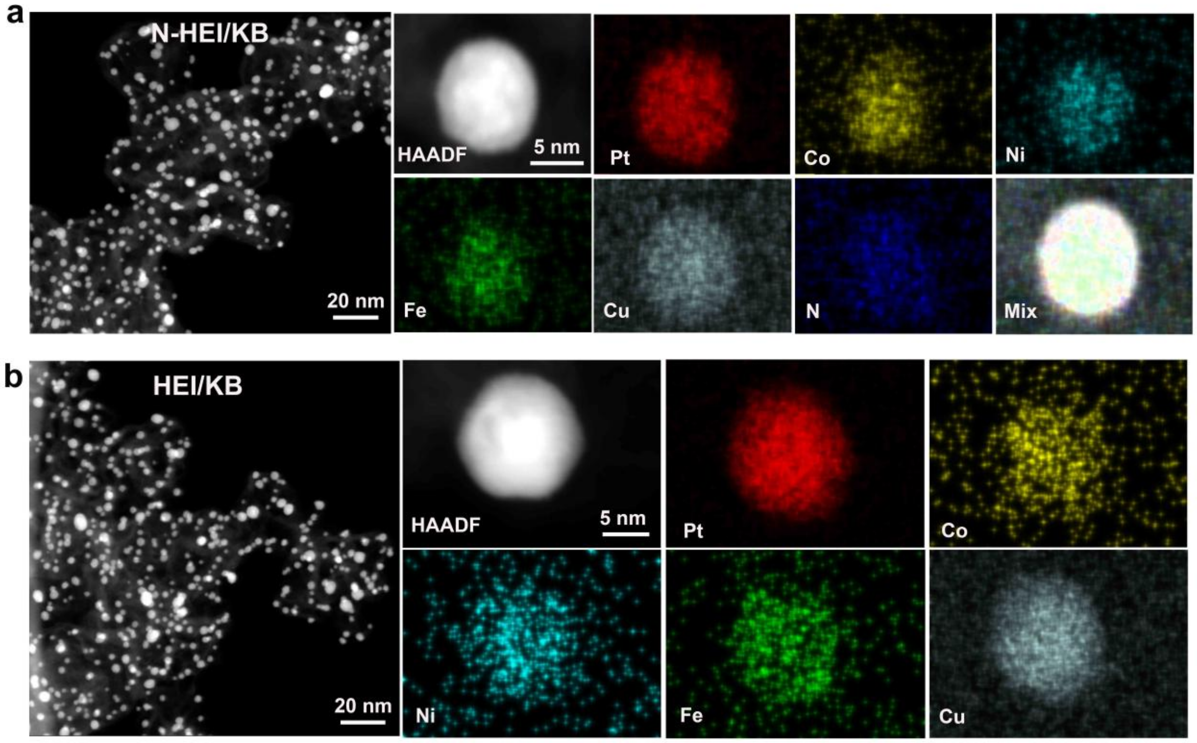

Supplementary Fig. 9 Structure and morphology of N-HEI/KB and HEI/KB catalyst after ADT cycles under RDE conditions. a. STEM-HAADF images and the EDS mappings of the N-HEI/KB catalyst after ADT 30k cycles. b. STEM-HAADF images and the EDS elemental mappings of the HEI/KB catalyst after ADT 30k cycles.

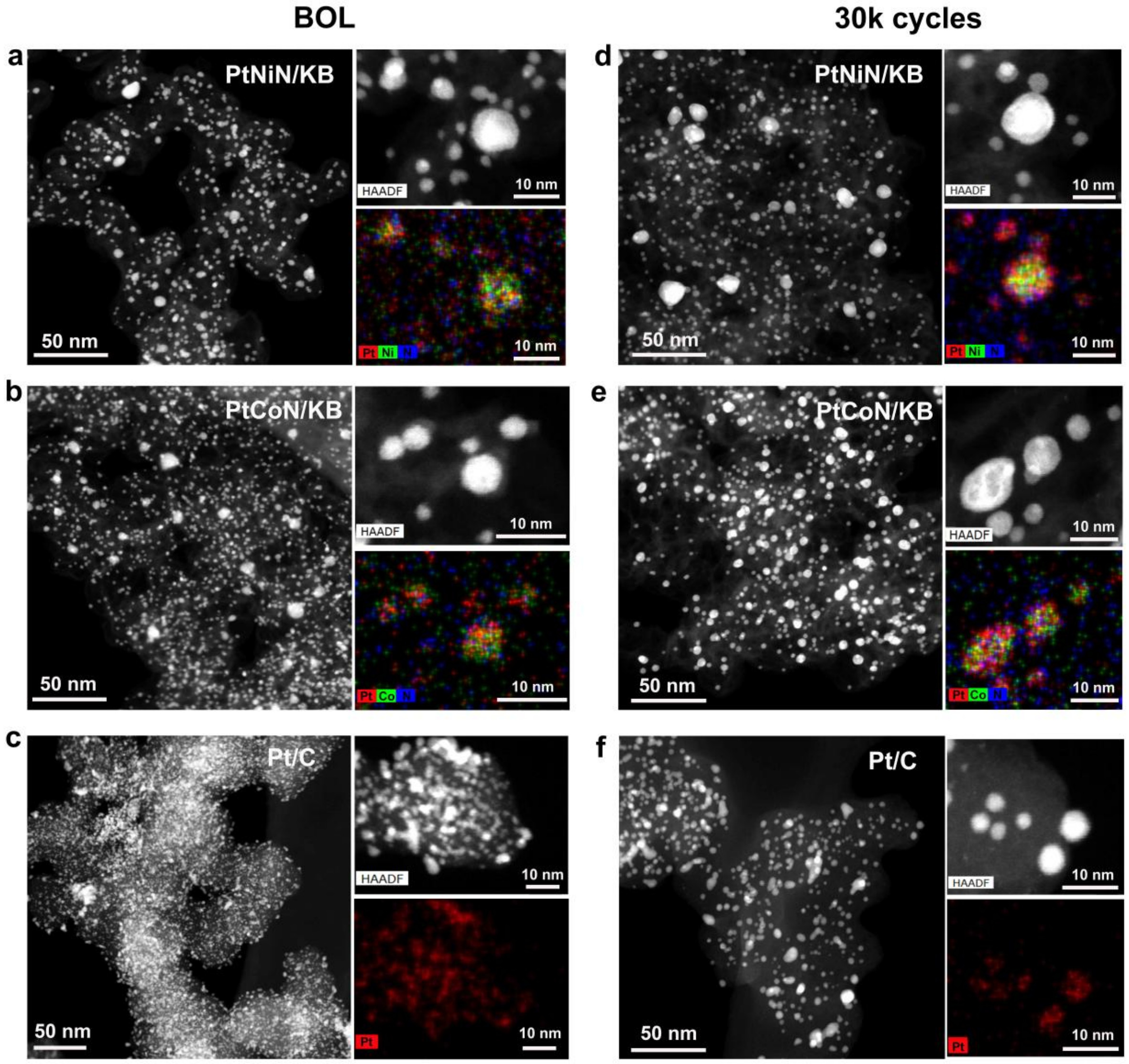

Supplementary Fig. 10 Structure and morphology of PtNiN/KB, PtCoN/KB, and Pt/C catalysts before and after ADT cycles in RDE conditions. a-c. STEM-HAADF images and the EDS mappings of PtNiN/KB, PtCoN/KB, and commercial Pt/C at BOL. d-f. STEM-HAADF images and the EDS mappings of PtNiN/KB, PtCoN/KB, and commercial Pt/C after ADT 30k cycles.

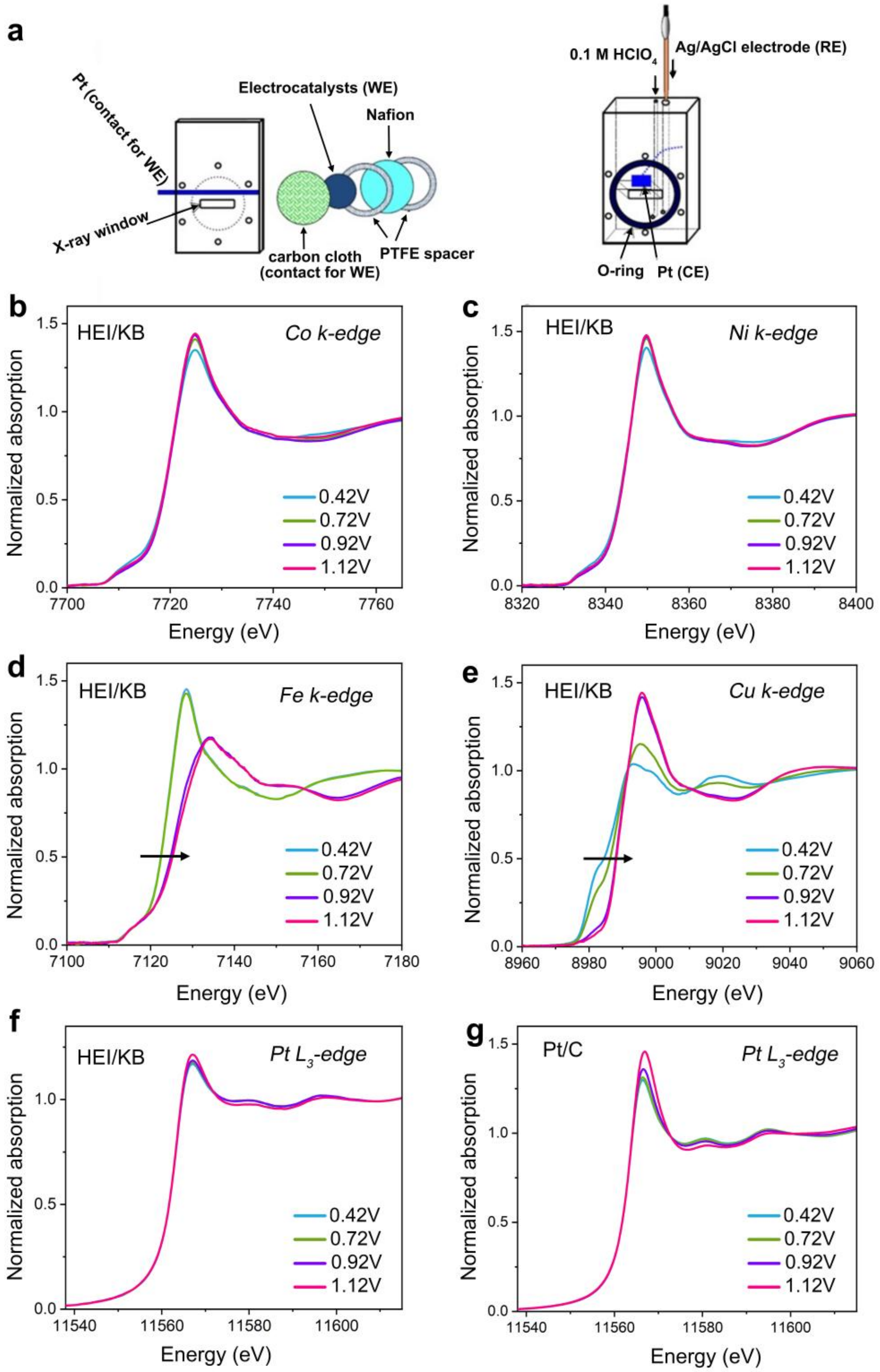

Supplementary Fig. 11 In situ XANES measurements of HEI/KB and commercial Pt/C. a. Schematic diagrams of in situ electrochemical cell. b-e. In situ normalized XANES spectra of N-Pt/HEA/C recorded at different potentials in fluorescence mode at the b. Co K-edge, c. Ni K-edge, d. Fe K-edge and e. Cu K-edge. f-g. Pt L_3-edge XANES spectra of f. HEI/KB and g. commercial Pt/C recorded at a series of applied potentials.

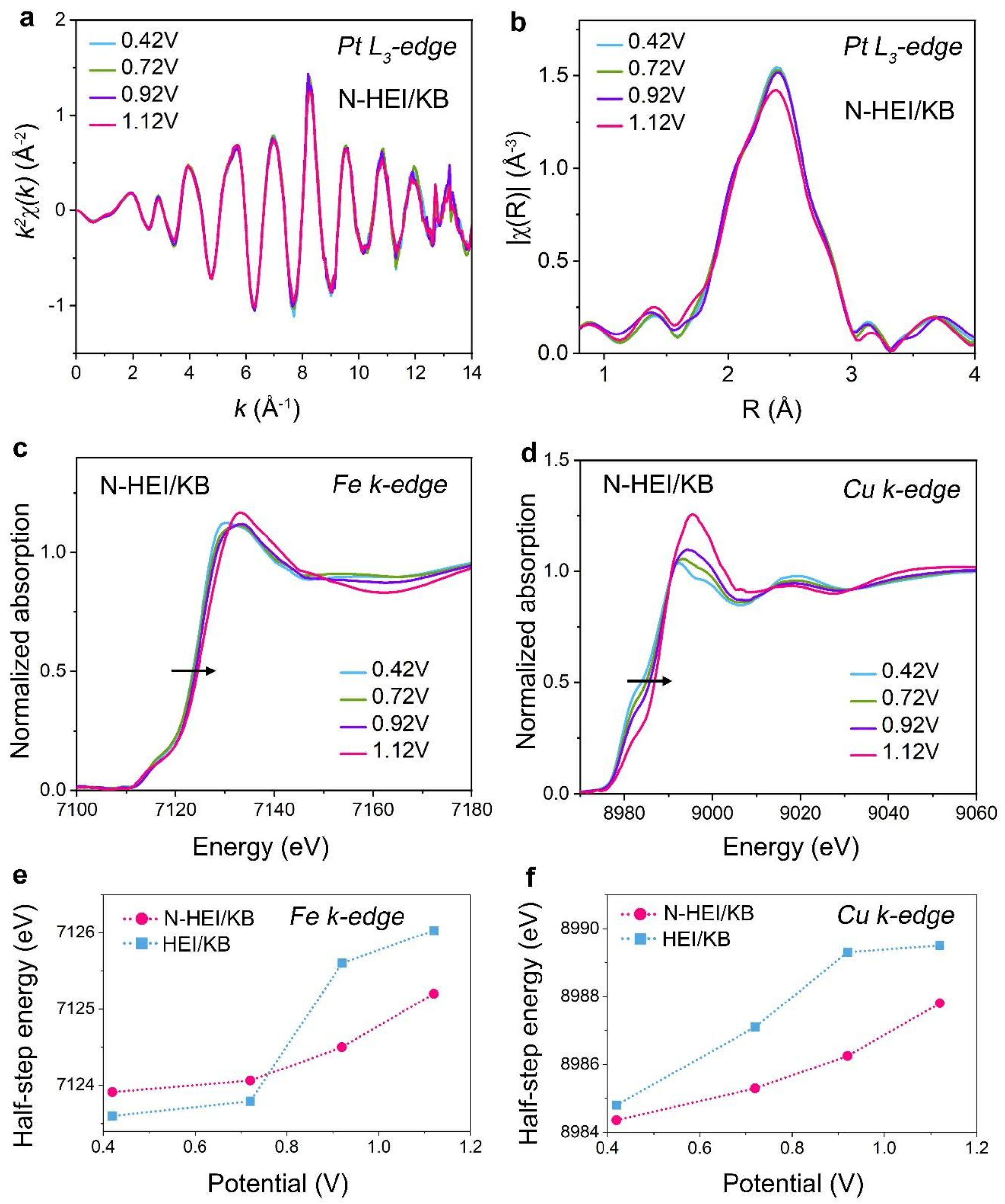

Supplementary Fig. 12 In-situ XANES measurements of N-HEI/KB. a. In situ Pt L_3-edge k-space (k^2-weighted) EXAFS. b. In situ Pt L_3-edge FT-EXAFS spectra of N-HEI/KB at different potentials. In situ normalized XANES spectra of N-HEI/KB recorded at different potentials in fluorescence mode at the c. Fe K-edge and d. Cu K-edge. The change in half-step energy in e. Fe K-edge and f. Cu K-edge with increasing potentials.

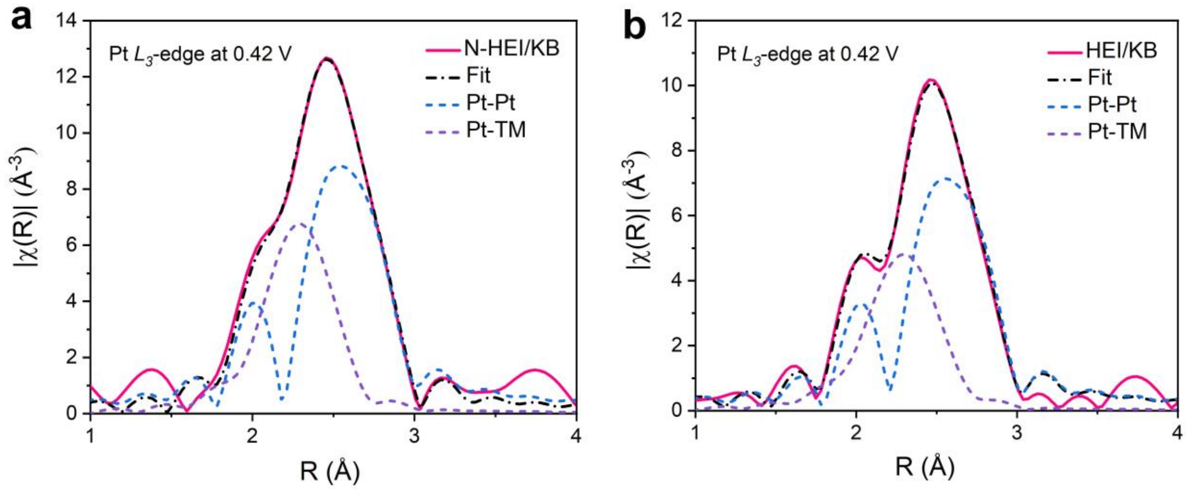

Supplementary Fig. 13 Pt L_3-edge FT-EXAFS spectra. a. N-HEI/KB and b. HEI/KB catalysts at 0.42 V with a first-shell fit together with Pt-Pt and Pt-TM contributions. The fitting of Pt L_3-edge spectra was performed by lumping Co, Ni, Fe, and Cu as one transition metal (TM). We used Ni for representing TM of Pt-TM fitting in FEFF calculation in Artemis.

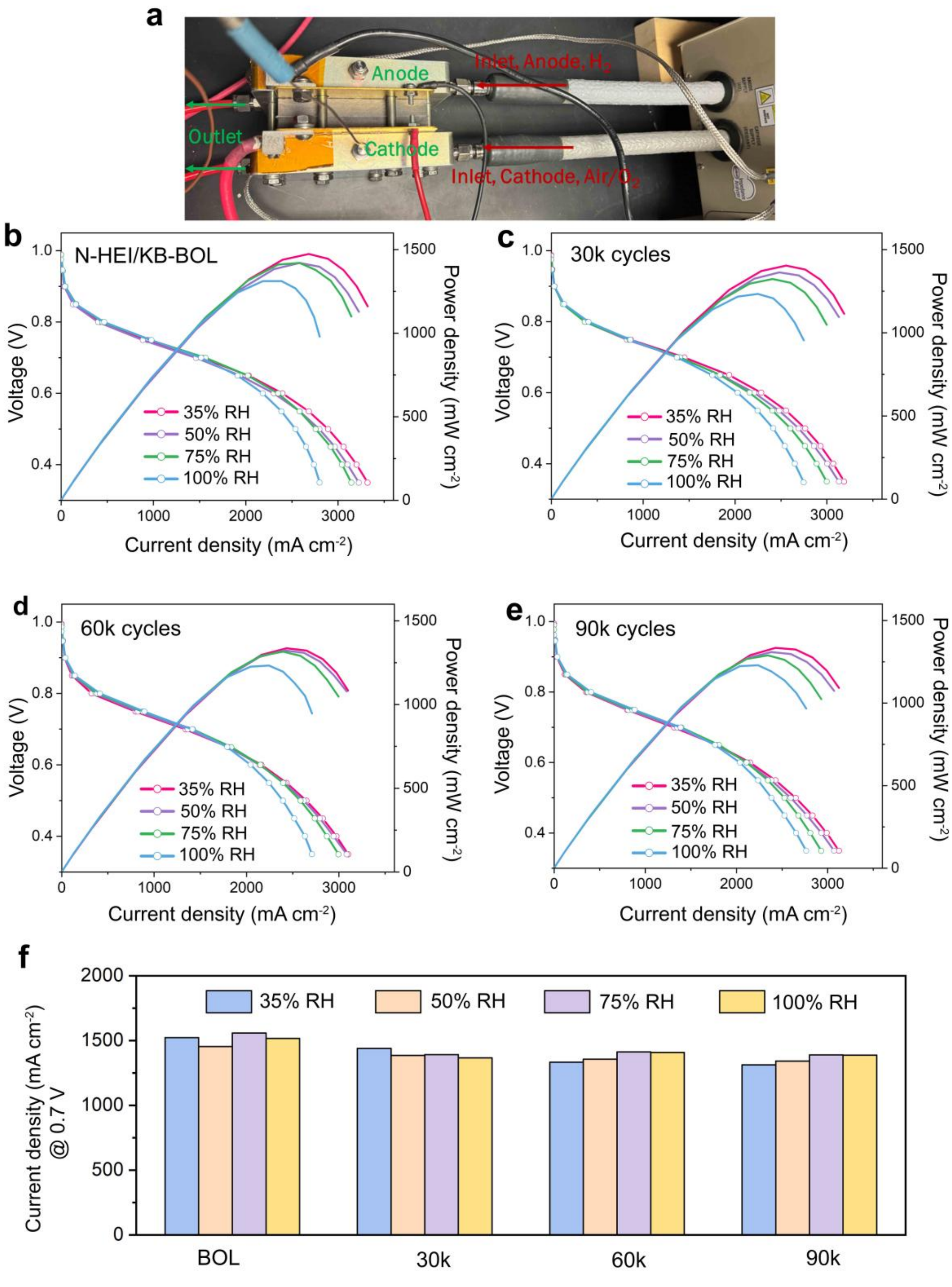

Supplementary Fig. 14 MEA performance of N-HEI/KB under various relative humidity at BOL and different cycles. a. Photograph of the fuel cell device. The I-V polarization curves and power density of the H_2-air fuel cell of the N-HEI/KB at b. BOL, c. 30k cycles, d. 60k cycles, and e. 90k cycles. f. The current density of N-HEI/KB at 0.7 V under various relative humidity (RH) at BOL and different cycles. MEA test conditions: 0.20 mg_{Pt} cm^{-2} (cathode Pt loadings), H_2/air (500/2000 sccm), 80 °C, and 250 kPa_{abs} pressure.

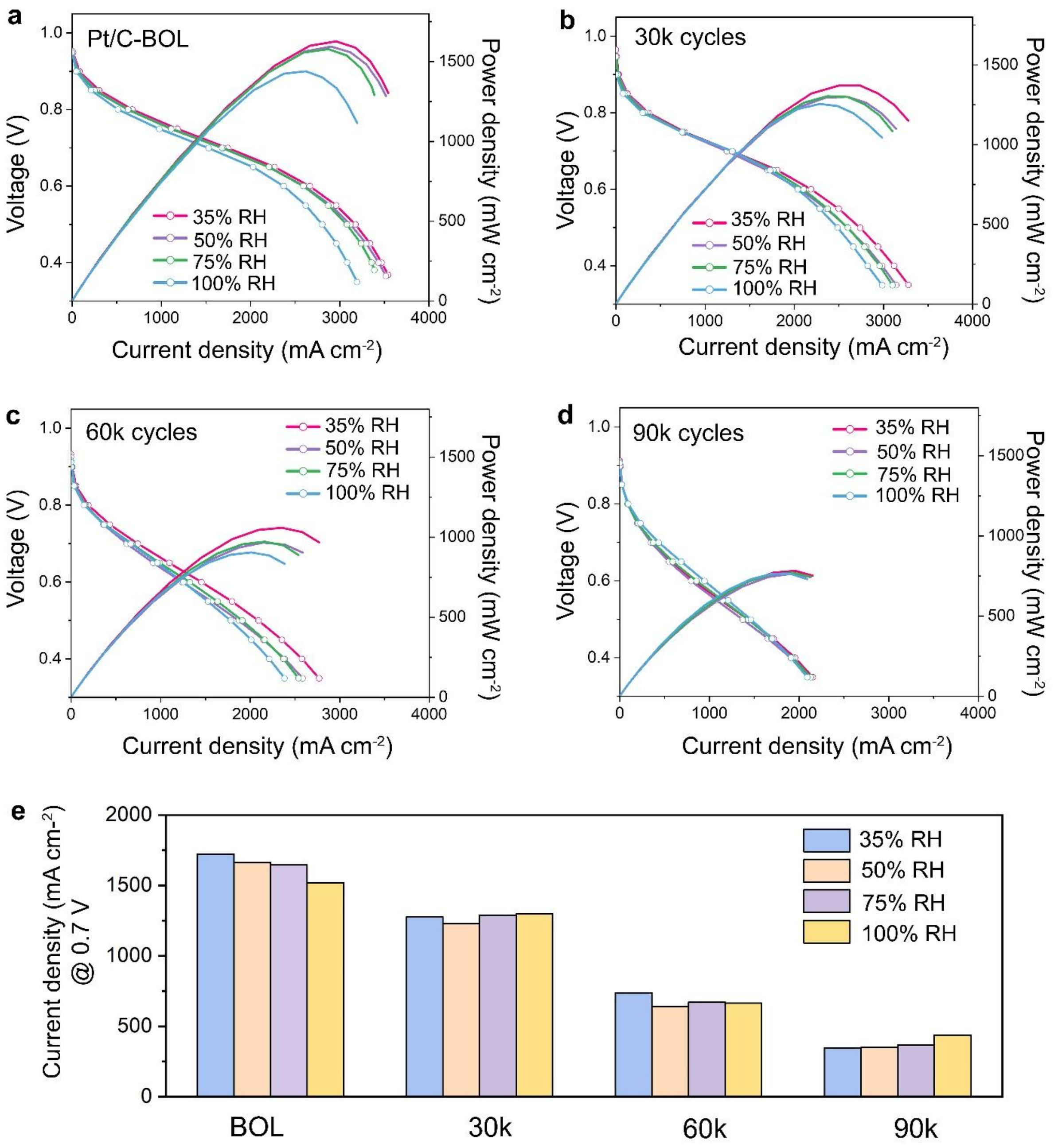

Supplementary Fig. 15 MEA performance of commercial Pt/C under various relative humidity at BOL and different cycles. The I-V polarization curves and power density of the H_2-air fuel cell of the commercial Pt/C at: a. BOL, b. 30k cycles, c. 60k cycles, and d. 90k cycles. e. The current density of N-HEI/KB at 0.7 V under various RH at BOL and different cycles. MEA test conditions: 0.20 mg_{Pt} cm^{-2} (cathode Pt loadings), H_2/air (500/2000 sccm), 80 °C, and 250 kPa_{abs} pressure.

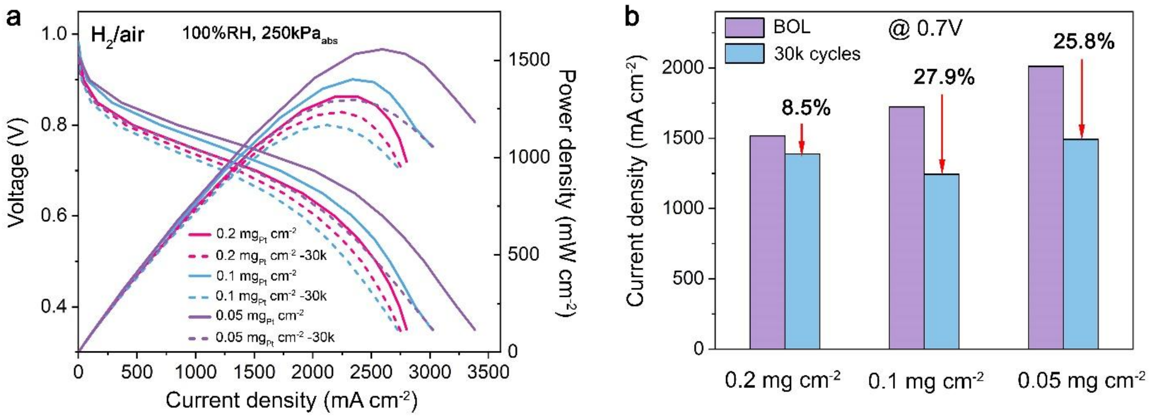

Supplementary Fig. 16 MEA performance of N-HEI/KB at different cathode loadings. a. H_2/air fuel cell performance of N-HEI/KB at different cathode loadings (0.2, 0.1, and 0.5 mg_{Pt} cm^{-2}) at BOL and 30k voltage cycles. b. Current densities of N-HEI/KB at 0.7 V at different cathode loadings at BOL and 30k voltage cycles.

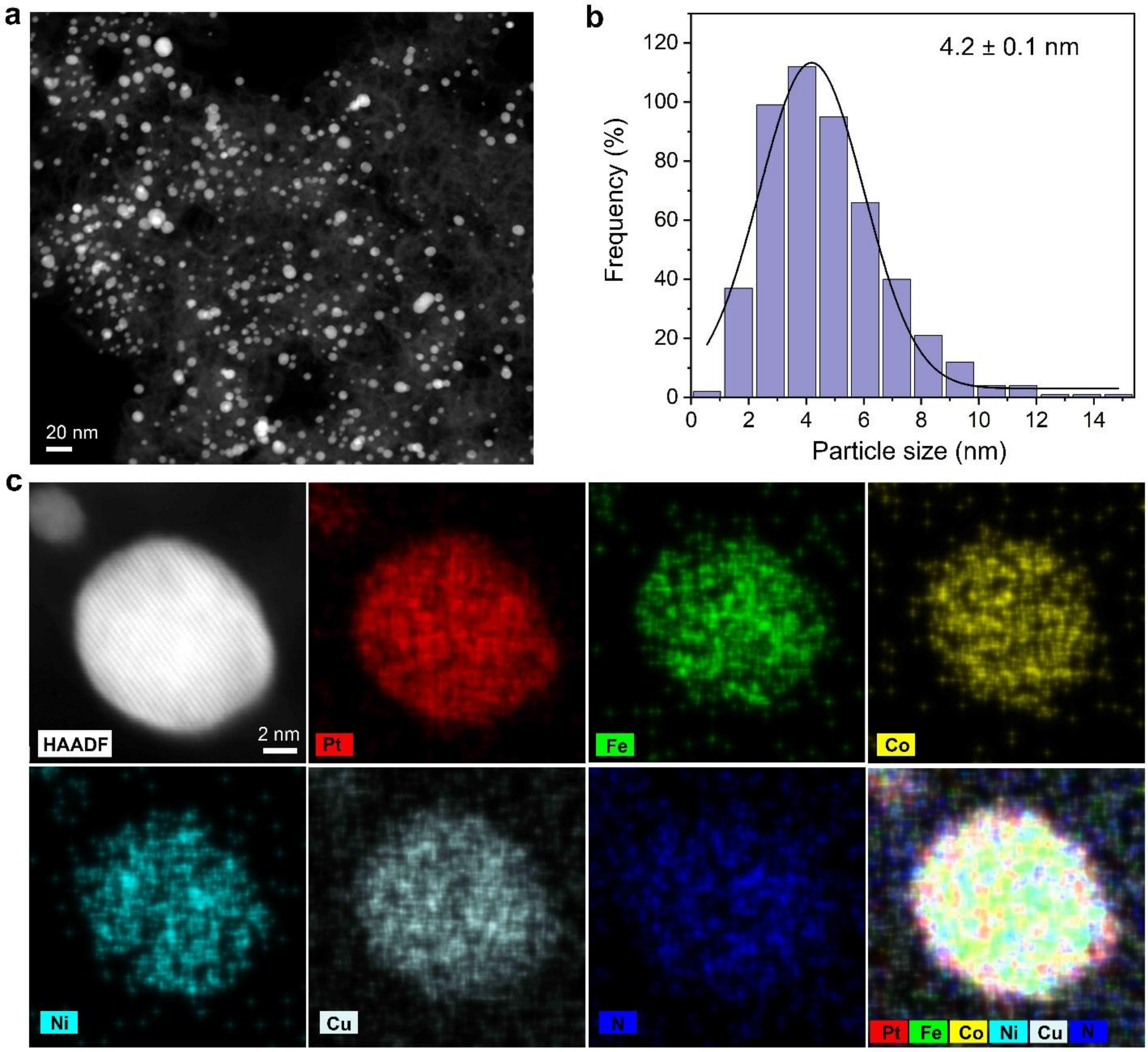

Supplementary Fig. 17 Structure and morphology of N-HEI/KB catalyst after ADT cycles in MEA testing. a. STEM-HAADF image and b. the particle size distribution of the N-HEI/KB catalyst after ADT 90k cycles. c. STEM-HAADF image and the EDS elemental mappings of the N-HEI/KB nanoparticles after ADT 90k cycles.

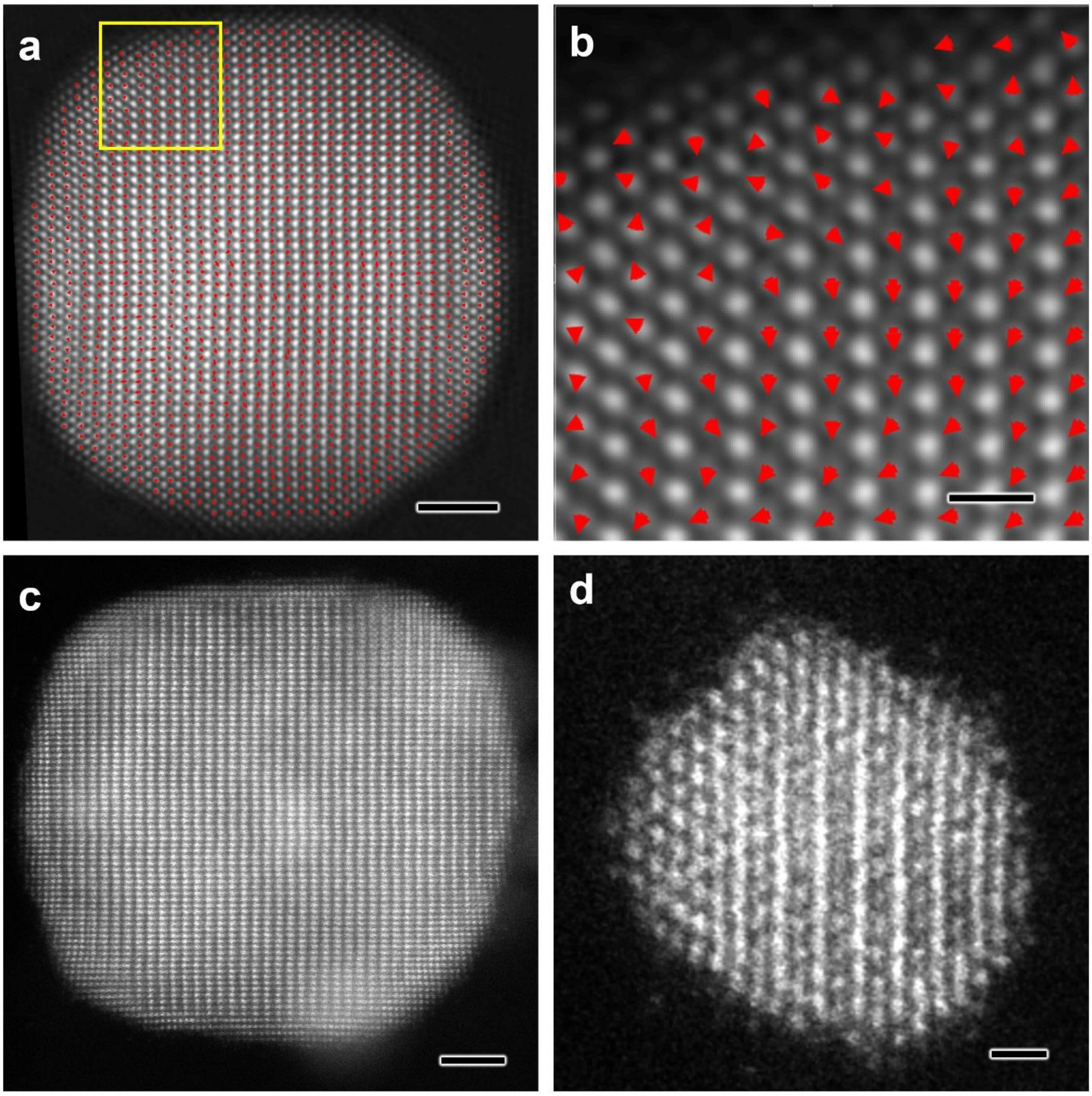

Supplementary Fig. 18 Atomic-resolution STEM-HAADF images of N-HEI/KB after MEA testing. a. The same atomic-resolution STEM-HAADF image as Fig. 3c (N-HEI nanoparticle after ADT 90k cycles in MEA testing) with TM displacement arrow map embedded. b. Magnified image from the yellow rectangle in (a). c-d. Additional STEM-HAADF images from N-HEI nanoparticle after ADT 90k cycles in MEA testing, showing multiple layers Pt shell. Scale bar 2 nm for (a,c) and 0.5 nm for (b,d).

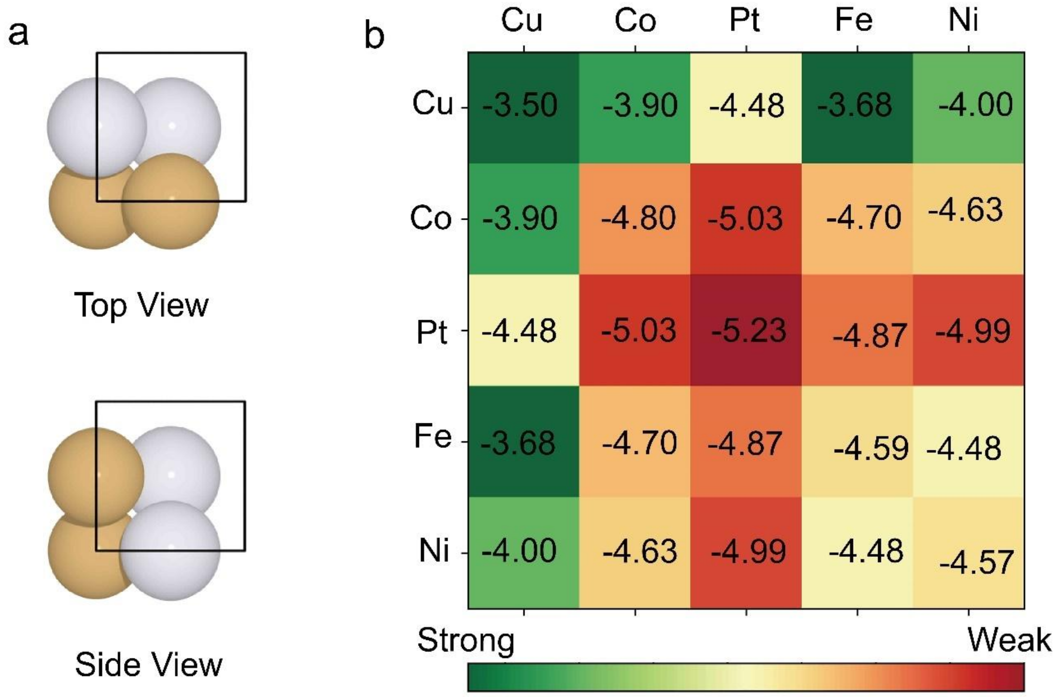

Supplementary Fig. 19 The model and cohesive energy of binary alloys. a. An illustration of the atomic model of $L1_0$-AB intermetallic structure. b. Binary alloy cohesive energy calculated by DFT.

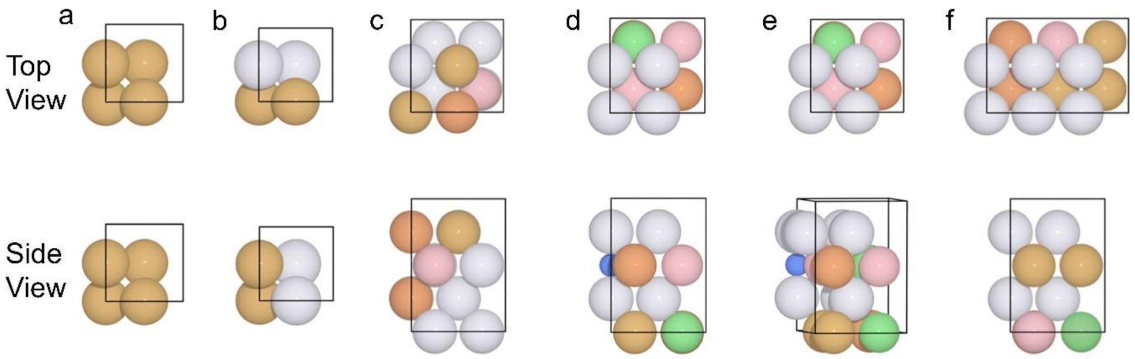

Supplementary Fig. 20 DFT calculated structure dataset for fine-tuning uMLIPs. a. pure transition metals, b. $L1_0$-AB intermetallic structures, c. HEA structures, d-e. (2x2x4) supercell of $L1_0$-HEI intermetallic structures with/without N interstitial, f. (2x3x4) supercell of $L1_0$-HEI intermetallic.

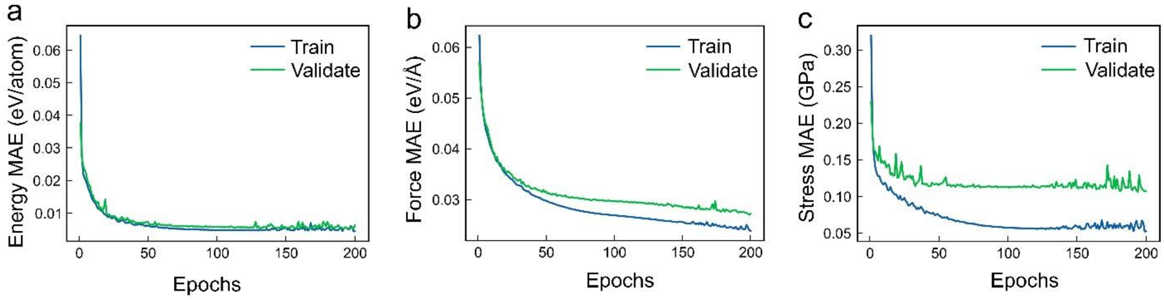

Supplementary Fig. 21 Performance of fine-tuned models in MAE with increasing training steps on train and validation set of the dataset.

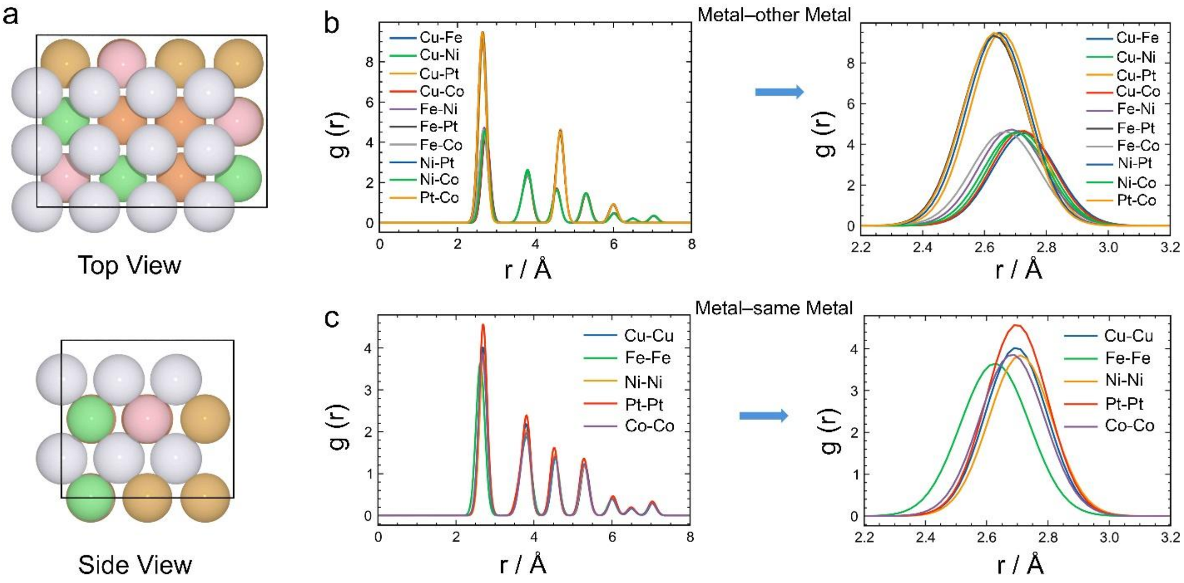

Supplementary Fig. 22 The atomic model and the average radial distribution functions of L1$_0$-HEI structures. a. An illustration of the 500 randomly generated atomic models of L1$_0$-HEI structures using fine-tuned uMLIPs. b-c. The average radial distribution functions (RDF) of these 500 L1$_0$-HEI structures.

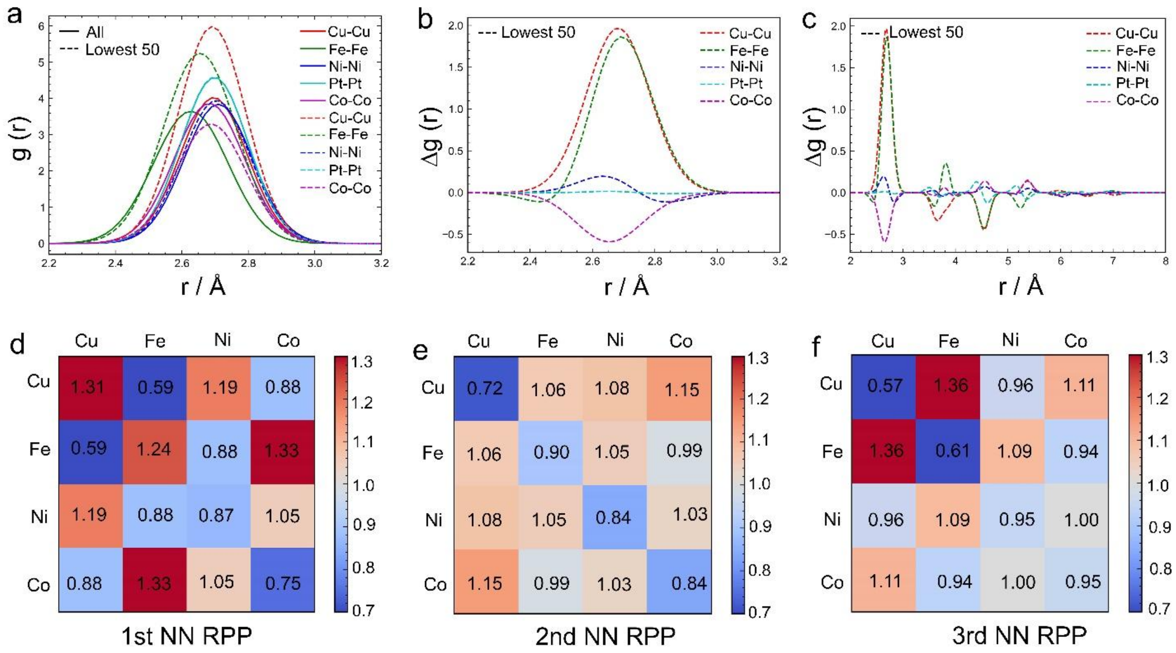

Supplementary Fig. 23 The RDF and RPP results of different $L1_0$-HEI structures. a. A comparison of the average RDF of 50 $L1_0$-HEI structures with the lowest potential energy predicted by the fine-tuned uMLIPs and all 500 $L1_0$-HEI structures. b-c. The normalized average RDF of these 50 $L1_0$-HEI structures. d-f. The relative pair probability (RPP) parameters up to 3rd nearest neighbor (NN) TM (TM=Cu, Fe, Ni, Co) pairs of these 50 $L1_0$-HEI structures.

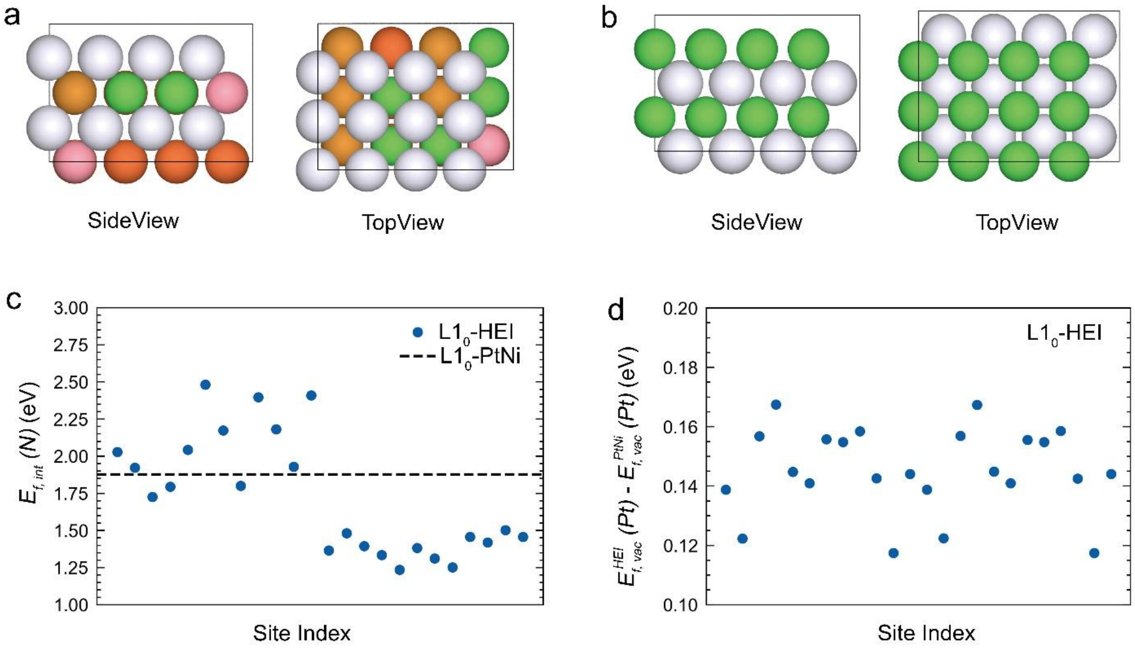

Supplementary Fig. 24 The formation energies of various N interstitial configurations and Pt vacancies in L1$_0$-HEI and L1$_0$-PtNi structures. a. L1$_0$-ordered structure of HEI. (Pt: gray, Co: pink, Ni: green, Fe: orange; Cu: brown). b. L1$_0$-ordered structure of PtNi (Pt: gray, Ni: green). c. N interstitial formation energy in L1$_0$-HEI and L1$_0$-PtNi model. d. The difference values of Pt vacancy formation energy between L1$_0$-HEI and L1$_0$-PtNi.

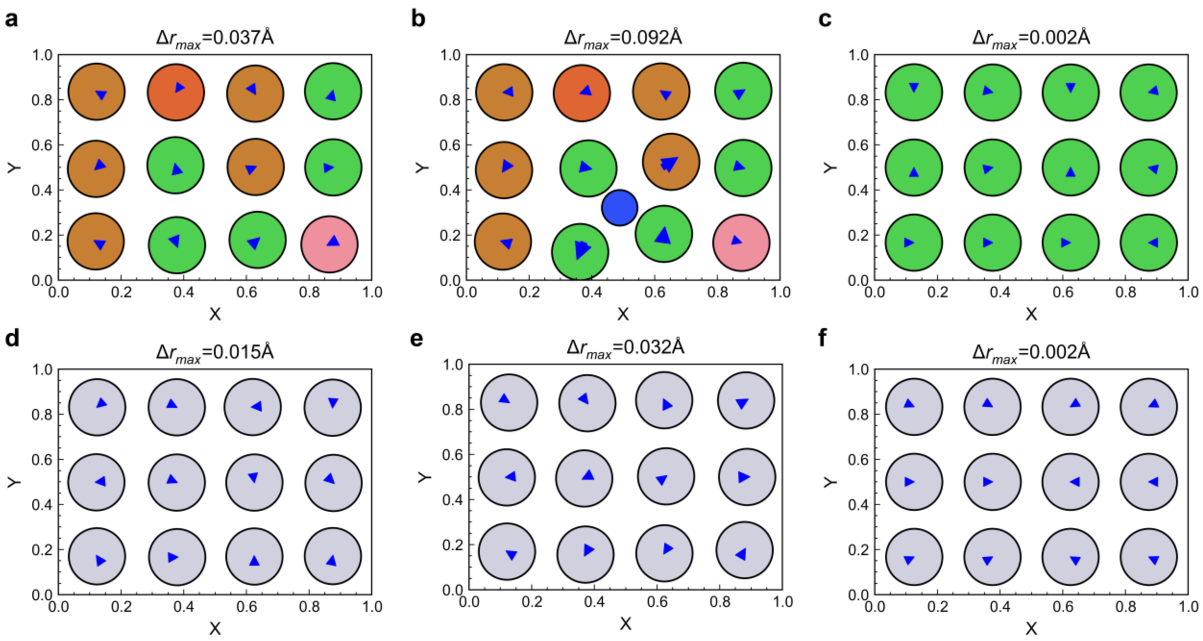

Supplementary Fig. 25 The mapping plot of site-dependent local atomic distortions in $L1_0$-HEI, $L1_0$-N-HEI and $L1_0$-PtNi intermetallics. The local lattice distortions in the TM layer are projected onto the (111) plane for a. $L1_0$-HEI, b. $L1_0$-N-HEI, and c. $L1_0$-PtNi, and those in the Pt layer for d. $L1_0$-HEI, e. $L1_0$-N-HEI, and f. $L1_0$-PtNi. The magnitude and direction of the local atomic distortion are indicated by arrows, and the max local atomic displacement are shown for each structure. Note that we used fractional coordinates and magnified the atomic displacements with the same scale for all plots to improve visualization. (Pt: gray, Co: pink, Ni: green, Fe: orange, Cu: brown, N: blue)

The site-dependent local atomic distortion is defined as the degree of atomic displacement from the ideal lattice sites $\Delta r_i' = r_i' - r_i$, where r_i and r_i' are the atomic coordinates of site *i* before and after lattice distortion, respectively. Note that the structural distortion of crystalline materials typically involves both lattice structure distortion (variations in the lattice structure) and the local atomic distortion (atomic displacements from ideal lattice sites). In our calculation, accounts only for local atomic distortion, as this is the primary contributor to sub-angstrom strain in this system.

Supplementary Table 1. Atomic ratios of HEI/KB and N-HEI/KB samples characterized by STEM-EDS.

	HEI/KB	N-HEI/KB
Pt	50.8	48.4
Co	11.1	10.4
Ni	13.4	12.1
Fe	12.2	11.6
Cu	12.5	11.8
N	----	5.7
Atomic ratio	$Pt_{51}Co_{11}Ni_{13}Fe_{12}Cu_{13}$	$Pt_{48}Co_{10}Ni_{12}Fe_{12}Cu_{12}N_6$

To estimate the errors, we measured multiple regions (≥10 regions per sample) and reported the average elemental compositions. The relative error of the STEM-EDS quantification was typically within ±2–4 at.% for the main metallic elements.

Supplementary Table 2. The element analysis of HEI/KB and N-HEI/KB by ICP-MS.

Sample	Pt (wt.%)	Co (wt.%)	Ni (wt.%)	Fe (wt.%)	Cu (wt.%)	Atomic ratio
HEI/KB	23.9	1.61	1.72	1.66	1.94	$Pt_{51}Co_{12}Ni_{12}Fe_{12}Cu_{13}$
N-HEI/KB	23.6	1.64	1.78	1.66	2.02	$Pt_{50}Co_{12}Ni_{13}Fe_{12}Cu_{13}$

The prepared solution of N-HEI/KB and HEI/KB samples were analyzed by inductively coupled plasma−mass spectroscopy (ICP-MS) to determine the contents of metals. Measurements were made with a PerkinElmer NexION 2000 ICP Mass Spectrometer calibrated with standards prepared from NIST traceable solutions. The sample was transferred to a pre-weighed quartz beaker and the mass was recorded. The samples was ignited in the furnace at 250°C (1 hr), 500°C (2 hr), and 700°C (overnight). After ignition, the beakers were cooled and then 1 mL of DDI, 3 mL of optima HNO_3, and 11 mL of optima HCl were added to each beaker. The samples were heated on the hotplate with a watchglass at 220°C for approximately 1 hr and then were added into solution. All samples were diluted to volume in a centrifuge tube. Nanoparticles were assumed to be gone after the ignition step. The data shown were calculated from the ICP-MS concentration data, the volume of the prepared solution, and the mass of sample taken.

Supplementary Table 3. Parameters and R-values obtained from Rietveld refinements for the N-HEI/KB and HEI/KB samples.

Samples	phase	Ratio (wt.%)	Lattice parameter		R_{wp}
			a=b	c	
N-HEI/KB	HEI	54.1	3.680±0.003 Å	2.710±0.002 Å	4.8%
	Pt	45.9	3.902±0.005 Å	3.902±0.005 Å	
HEI/KB	HEI	59.4	3.675±0.003 Å	2.710±0.002 Å	4.6%
	Pt	40.6	3.899±0.006 Å	3.899±0.006 Å	

Supplementary Table 4. The $E_{1/2}$, MA, ECSA (H_{upd}), and SA of N-HEI/KB at different cycle RDE tests.

Catalysts	$E_{1/2}$ (mV)	MA @ 0.9 V ($A\ mg_{Pt}^{-1}$)	ECSA ($m^2\ g^{-1}$)	SA @ 0.9 V ($mA\ cm^{-2}$)
BOL	925	1.45	61.2	2.37
10k	927	1.51	58.5	2.58
20k	927	1.54	58.0	2.66
30k	920	1.19	56.4	2.05
60k	918	1.04	55.2	1.88
90k	915	0.98	54.4	1.80

Supplementary Table 5. The $E_{1/2}$, MA, ECSA (H_{upd}), and SA of HEI/KB, PtNiN/KB, PtCoN/KB, and commercial Pt/C at different ADT cycles.

Catalysts	Cycles	$E_{1/2}$ (mV)	MA @ 0.9 V (A mg_{Pt}^{-1})	ECSA (m^2 g^{-1})	SA@0.9V (mA cm^{-2})
HEI/KB	0	919	1.10	54.8	2.01
	30 k	911	0.81	51.7	1.57
	loss	-8	-26.4%	-5.7%	-21.9%
PtNiN/KB	0	930	1.23	64.6	1.90
	30k	915	0.83	55.7	1.49
	loss	-15	-32.5%	-13.8%	-21.6%
PtCoN/KB	0	924	0.88	72.7	1.21
	30 k	903	0.53	57.5	0.92
	loss	-21	-39.8%	-20.9%	-24.0%
Pt/C	0	900	0.24	83.3	0.29
	30 k	882	0.15	62.2	0.24
	loss	-18	-37.5%	-25.3%	-32.3%

Supplementary Table 6. Comparison of the electrocatalytic performance of the advanced catalysts reported in recent literatures for oxygen reduction reaction in 0.1 M $HClO_4$ solutions.

Catalysts	MA @ 0.9 V (A mg_{PGM}^{-1})	MA loss (ADT cycles)	Decay of $E_{1/2}$ (mV)	Reference
**N-HEI/KB**	**1.45**	**17.9% (30k)** **32.4% (90k)**	**5 (30k)** **10 (90k)**	**This work**
PtIrFeCoCu-HEI/C	1.29	----	9 (60k)	Ref[2]
PtFeCoNiCuZn-HEI	2.403	5.9% (10k)	~1 (10k)	Ref[3]
PtPdCoFeNi HEA/C	1.17	23.6% (50k)	6 (50k)	Ref[4]
PtNiFeCuCoZn/PC	0.5	2% (5k)	1 (5k)	Ref[5]
$Pt(FeCoNiCuZn)_3/C$	0.7	2.9% (30k)	~1 (30k)	Ref[6]
$Pt_4FeCoCuNi$	3.78	26% (30k)	7 (30k)	Ref[7]
N-Pt/HEA/C	1.34	20.9% (30k)	8 (30k)	Ref[8]
$L1_0$-Pt_2CuGa/C	1.39	18.7% (10k)	8 (30k)	Ref[9]
PtCuCo@Co-N-C	1.14	----	19 (40k)	Ref[10]
O-$Fe_3Pt/Ti_{0.5}Cr_{0.5}N$	0.673	9.7% (5k)	6 (5k)	Ref[11]
Gd-O-Pt_3Ni	1.54	27.9% (70k)	8 (70k)	Ref[12]
FePt@PtBi	0.96	18% (30k)	----	Ref[13]

Supplementary Table 7. Element loading on the carbon support for different catalysts at BOL and after 30k cycles based on the average of 10 STEM-EDS results.

Samples	BOL		30k cycles		Element loss	
	Pt (wt.%)	M* (wt.%)	Pt (wt.%)	M* (wt.%)	Pt (%)	M*(%)
N-HEI/KB	22.4	6.7	21.8	5.8	-2.7%	-13.4%
HEI/KB	22.1	6.2	21.4	4.7	-3.2%	-24.2%
PtNiN/KB	22.6	6.5	20.3	3.6	-10.2%	-44.6%
PtCoN/KB	23.5	5.8	21.2	1.8	-9.8%	-69.0%
Pt/C	46.1	----	34.7	----	-24.7%	----

*M refers to the transition metals in different Pt-based catalysts. Specifically, M represents the total loading of transition metals (Co/Fe/Ni/Cu) in N-HEI/KB and HEI/KB.

Supplementary Table 8. Fitting results of bonding distances (R) and coordination numbers (CN) of Pt-Pt and Pt-TM pairs of the N-HEI/KB and HEI/KB catalysts.

	R(Pt-Pt) (Å)	R(Pt-TM) (Å)	CN(Pt-Pt)	CN(Pt-TM)
N-HEI/KB	2.710 (±0.005)	2.634 (±0.005)	6.9 (±0.8)	3.2 (±0.6)
HEI/KB	2.698 (±0.003)	2.632 (±0.005)	5.0 (±0.4)	3.0 (±0.4)

Supplementary Table 9. Summary of MEA performance of the recently reported Pt-based electrocatalysts.

	Cathode loading (mg_{Pt} cm^{-2})	Current density @ 0.7 V (A cm^{-2})		Voltage loss @0.8 A cm^{-2} (mV)	Test Conditions (temperature, pressure)	Reference
		BOL	EOL			
**N-HEI/KB**	**0.2**	**1.52**	**1.39 (90k)**	**9 (90k)**	**80°C, 250 kPa_{abs}**	**This work**
Pt (40wt%)/Mn-N-C	0.25	1.41	1.20 (150k)	----	80°C, 250 kPa_{abs}	Ref[14]
$L1_2$-Pt_3Co@Mn_{SA}-NC	0.2	1.75	1.43 (90k)	30* (90k)	80°C, 250 kPa_{abs}	Ref[15]
PtCo/KB-NH_2	0.2	1.31	0.83 (90k)	30 (30k)	80°C, 150 kPa_{abs}	Ref[16]
Gd-O-Pt_3Ni	0.1	1.24	0.81 (50k)	19 (40k)	80°C, 200 kPa_{abs}	Ref[12]
i-CoPt/KB	0.1	1.5*	1.25*(30k)	29 (30k)	80°C, 250 kPa_{abs}	Ref[17]
PtCo/Gnp	0.06	1.22*	1.06* (30k)	23.5 (30k)	80°C, 150 kPa_{abs}	Ref[18]
Pt_3Co/FeN_4-C	0.1	1.05*	0.8* (30k)	22 (30k)	80°C, 150 kPa_{abs}	Ref[19]
STG-PtCo	0.025	1.2*	1.0* (30k)	21 (30k)	80°C, 150 kPa_{abs}	Ref[20]
$L1_0$-PtZn	0.1	0.96	0.91 (30k)	10 (30k)	80°C, 150 kPa_{abs}	Ref[21]
PtNi/C3	0.1	1.0*	0.7* (30k)	16 (30k)	94°C, 250 kPa_{abs}	Ref[22]
40%$L1_0$-$Pt_{50}Ni_{35}Ga_{15}$/C	0.2	1.67	1.33 (90k)	25 (90k)	80°C, 250 kPa_{abs}	Ref[23]
PtNiMoAu NWs/C	0.1	0.93	0.72 (30k)	25 (30k)	80°C, 150 kPa_{abs}	Ref[24]
N-Pt/HEA/C	0.2	1.64	1.44 (30k)	18 (30k)	80°C, 250 kPa_{abs}	Ref[8]

*Data extracted from plots in the literatures.

Supplementary Table 10. Current density of N-HEI/KB and commercial Pt/C at 0.7 V.

Current density @0.7V (mA cm^{-2})	N-HEI/KB				Pt/C			
	35% RH	50% RH	75% RH	100% RH	35% RH	50% RH	75% RH	100% RH
BOL	1523	1455	1559	1517	1723	1662	1647	1520
30k	1440	1385	1392	1367	1278	1229	1290	1298
60k	1334	1356	1412	1408	737	639	670	665
90k	1312	1342	1390	1388	345	350	366	436
Loss	-13.9%	-7.8%	-10.8%	-8.5%	-80.0%	-78.9%	-77.8%	-71.3%

Supplementary Table 11. The test MAE of fine-tuned uMLIPs for single-point calculations.

MAE	Energy (eV/atom)	Force (eV/Å)	Stress (Gpa)
M3GNET (Fine-tuned)	0.00313	0.0283	0.0593
M3GNET	0.337	0.0567	2.288

Supplementary Table 12. The test MAE of fine-tuned uMLIPs for prediction relaxed structures.

MAE	Energy (eV/atom)	Force (eV/Å)	Stress (Gpa)	Lattice (Å)	Volume (Å^3)
M3GNET (Fine-tuned)	0.00279	0.0141	0.121	0.00883	0.243
M3GNET	0.327	0.0141	0.121	0.0325	21.279

Supplementary References

1 Coelho, A. A. TOPAS and TOPAS-Academic: an optimization program integrating computer algebra and crystallographic objects written in C++. *J. Appl. Cryst.* **51**, 210-218 (2018).

2 Feng, G. *et al.* Engineering structurally ordered high-entropy intermetallic nanoparticles with high-activity facets for oxygen reduction in practical fuel cells. *J. Am. Chem. Soc.* **145**, 11140-11150 (2023).

3 Chen, T. *et al.* An ultrasmall ordered high-entropy intermetallic with multiple active sites for the oxygen reduction reaction. *J. Am. Chem. Soc.* **146**, 1174-1184 (2024).

4 Yu, Y. *et al.* High-entropy alloy nanoparticles as a promising electrocatalyst to enhance activity and durability for oxygen reduction. *Nano Res.* **15**, 7868-7876 (2022).

5 Zhang, W., Feng, X., Mao, Z. X., Li, J. & Wei, Z. Stably immobilizing sub-3 nm high-entropy Pt alloy nanocrystals in porous carbon as durable oxygen reduction electrocatalyst. *Adv. Funct. Mater.* **32** (2022).

6 Zhang, Q. *et al.* High-entropy $L1_2$-$Pt(FeCoNiCuZn)_3$ intermetallics for ultrastable oxygen reduction reaction. *J. Energy Chem.* **86**, 158-166 (2023).

7 Wang, Y. *et al.* Ordering-dependent hydrogen evolution and oxygen reduction electrocatalysis of high-entropy intermetallic $Pt_4FeCoCuNi$. *Adv. Mater.* e2302067 (2023).

8 Zhao, X. *et al.* Multiple Metal-nitrogen bonds synergistically boosting the activity and durability of high-entropy alloy electrocatalysts. *J. Am. Chem. Soc.* **146**, 3010-3022 (2024).

9 Liu, X. *et al.* Inducing covalent atomic interaction in intermetallic Pt alloy nanocatalysts for high-performance fuel cells. *Angew. Chem. Int. Ed.* **62**, e202302134 (2023).

10 Huang, L. *et al.* Boosting oxygen reduction via integrated construction and synergistic catalysis of porous platinum alloy and defective graphitic carbon. *Angew. Chem. Int. Ed.* **60**, 25530-25537 (2021).

11 Liu, Q. *et al.* Structurally ordered Fe_3Pt nanoparticles on robust nitride support as a high performance catalyst for the oxygen reduction reaction. *Adv. Energy Mater.* **9** (2019).

12 Yang, L. *et al.* Rare earth evoked subsurface oxygen species in platinum alloy catalysts enable durable fuel cells. *Angew. Chem. Int. Ed.* **63**, e202315119 (2024).

13 Guan, J. *et al.* Intermetallic FePt@PtBi core-shell nanoparticles for oxygen reduction electrocatalysis. *Angew. Chem. Int. Ed.* **60**, 21899-21904 (2021).

14 Zeng, Y. *et al.* Pt nanoparticles on atomic-metal-rich carbon for heavy-duty fuel cell catalysts: durability enhancement and degradation behavior in membrane electrode sssemblies. *ACS Catal.* **13**, 11871-11882 (2023).

15 Zeng, Y. *et al.* Regulating catalytic properties and thermal stability of Pt and PtCo intermetallic fuel-cell catalysts via strong coupling effects between single-metal site-rich carbon and Pt. *J. Am. Chem. Soc.* **145**, 17643-17655 (2023).

16 Gong, Q. *et al.* Amino-tethering synthesis strategy toward highly accessible sub-3-nm $L1_0$-PtM catalysts for high-power fuel cells. *Matter* **6**, 963-982 (2023).

17 Yoo, T. Y. *et al.* Scalable production of an intermetallic Pt–Co electrocatalyst for high-power proton-exchange-membrane fuel cells. *Energy Environ. Sci.* **16**, 1146-1154 (2023).

18 Zhao, Z. *et al.* Graphene-nanopocket-encaged PtCo nanocatalysts for highly durable fuel cell operation under demanding ultralow-Pt-loading conditions. *Nat. Nanotechnol.* **17**, 968-975 (2022).

19 Qiao, Z. *et al.* Atomically dispersed single iron sites for promoting Pt and Pt_3Co fuel cell

catalysts: performance and durability improvements. *Energy Environ. Sci.* **14**, 4948-4960 (2021).

20 Song, T. W. *et al.* Small molecule-assisted synthesis of carbon supported platinum intermetallic fuel cell catalysts. *Nat. Commun.* **13**, 6521 (2022).

21 Liang, J. *et al.* Biaxial strains mediated oxygen reduction electrocatalysis on fenton reaction resistant $L1_0$-PtZn fuel cell cathode. *Adv. Energy Mater.* **10,** 2000179 (2020).

22 Zhao, Z. *et al.* Tailoring a three-phase microenvironment for high-performance oxygen reduction reaction in proton exchange membrane fuel cells. *Matter* **3**, 1774-1790 (2020).

23 Liang, J. *et al.* Metal bond strength regulation enables large-scale synthesis of intermetallic nanocrystals for practical fuel cells. *Nat. Mater.* **23,** 1259-1267 (2024).

24 Gao, L. *et al.* Identifying the distinct roles of dual dopants in stabilizing the platinum-nickel nanowire catalyst for durable fuel cell. *Nat. Commun.* **15**, 508 (2024).
