# Supplementary material for: Sub-angstrom strain in high-entropy intermetallic boosts the oxygen reduction reaction in fuel cell cathodes
Source: Nat Commun. 2025 Aug 14;16:7547. doi: 10.1038/s41467-025-62725-7 (PMC12354711; doi:10.1038/s41467-025-62725-7)
Supplement: Supplementary file 2 — Description of Additional Supplementary Data 1 [file 41467_2025_62725_MOESM2_ESM.docx]

**Description of Additional Supplementary Files**

Supplementary Data 1

Description: Optimized structures of L1_0_-HEI configurations.
